# Development of Multifunctional Cosmetic Cream Using Bioactive Materials from *Streptomyces* sp. T65 with Synthesized Mesoporous Silica Particles SBA-15

**DOI:** 10.3390/antiox9040278

**Published:** 2020-03-26

**Authors:** Ram Hari Dahal, Tuan Manh Nguyen, Dong Seop Shim, Joon Young Kim, Jangyul Lee, Jaisoo Kim

**Affiliations:** 1 Department of Life Science, College of Natural Sciences, Kyonggi University, Suwon, Gyeonggi-Do 16227, Korea; ramhari.dahal@gmail.com (R.H.D.); tuannm@tnu.edu.vn (T.M.N.); 2 Institute of Life Sciences, Thai Nguyen University of Agriculture and Forestry, Quyet Thang, Thai Nguyen 250-000, Vietnam; 3 Innogene, Co., #301 Woolim E-biz Center1, 28, Digital-ro 33-gil, Guro-gu, Seoul 08337, Korea; dshim@innogene.com; 4 Gyeonggi Small & Medium Business Growth Accelerating Center, 304ho, Suseong-ro 8, Gwonseon-gu, Suwon 16426, Korea; ooridle@gmail.com (J.Y.K.); zzangbaw@hanmail.net (J.L.)

**Keywords:** antioxidant, cytotoxicity, anti-tyrosinase, anti-aging, antimicrobial, mesoporous silica particles, cosmeceutical formulations, aesthetic application

## Abstract

Various cosmetics having a single function are increasingly being used, but cosmetics having multifunctional activities remain limited. We aimed to develop a multifunctional cosmetic cream having antioxidant, anti-tyrosinase, anti-aging and antimicrobial activities. Antimicrobial activities were performed by disc-diffusion method. Cell toxicity and cell proliferations were evaluated in a 96-well plate with different cell lines such as HaCaT, RAW264.7, CCD-986Sk, B16F1, and B16F10. Mushroom tyrosinase inhibition, elastase inhibition, and 1,1-diphenyl-2-picrylhydrazyl (DPPH) radical scavenging activities were evaluated and IC_50_ was calculated. Mesoporous silica particle was synthesized using Pluronic P123 and tetraethyl ortho-silicate (TEOS). Facial pictures were captured by VISIA-CR (Facial Imaging System for Clinical Research). Roughness of image was analysed by PRIMOS software and brightness of image was analyzed by Chromameter CR-400. The crude product of strain T65 inhibited the different human pathogenic bacteria such as *Bacillus subtilis*, *Escherichia coli*, *Propionibacterium acnes*, *Staphylococcus aureus*, *Pseudomonas aeruginosa*, and *Staphylococcus epidermidis*. The IC_50_ of T65 crude product for mushroom tyrosinase, elastase, and DPPH radical scavenging activities were 58.73, 14.68, and 6.31 µg/mL, respectively. T65 crude product proliferated collagen type I in CCD-986Sk cell up to 145.91% ± 9.11% (mean ± SD; mean of 24, 48, and 72 h) at 250 pg/mL. Synthesized mesoporous particles (SBA-15) confirmed the sustainable performance by control-release for three days. Formulated functional cosmetic cream containing T65 embedded SBA-15, significantly decreased the skin roughness by 4.670% and increased the skin brightness by 0.472% after application of 4 weeks. T65 crude product inhibited both Gram-positive and Gram-negative pathogens. Synthesized mesoporous particle, SBA-15, confirmed the physiologically active substance was released in sustainable release condition. T65 crude product showed impeccable antimicrobial, antioxidant, anti-aging, and whitening activities with non-cytotoxic effects to different cell lines related to the human skin.

## 1. Introduction

The skin is the largest organ and outer covering of the human body. The visual appearance of the skin allows for estimation of age, gender, health, attractiveness, and beauty [1,2]. Cosmetic products are extensively used for enhancing the appearance of the skin and to reduce skin aging. In addition, topical application of cosmetics demonstrates how to increase attractiveness by manipulating factors of beauty associated with facial contrast [3,4]. Cosmetic industries are constantly searching for novel and natural bioactive materials with anti-aging, antioxidant, anti-tyrosinase, and antimicrobial properties for cosmeceutical formulations to improve skincare approaches [5,6].

Skin aging is a complex biological process caused by various intrinsic and extrinsic factors that lead to physiological dysfunction and loss of the structural integrity of the skin. Intrinsic aging, generally known as natural aging (chronologic aging) of the skin is related to hormonal changes based on age, whereas extrinsic aging is associated with movement of the muscle, pollution, nicotine, exposure to solar radiation, caffeine, temperature, lifestyle such as diet (nutrition), lack of sleep, stress, and other health conditions [7,8,9]. Elastin helps skin to return to its original position after movement, whereas elastase produced in acinar cells degrades the elastin and leads to skin aging [9]. Anti-elastase inhibits the elastase and retains elastin in its initial status, which prevents skin from aging. Cosmetic or skin care products with anti-aging properties positively affect skin aging [10,11,12,13].

Free radicals are usually generated during cellular metabolism. Reactive oxygen species (ROS) and reactive nitrogen species (RNS), such as anion radical, superoxide, peroxide, hydroxyl radical, nitric oxide (NO), peroxynitrile, and hypochlorous acid are responsible for oxidative damage to the cells, lipids, proteins, and DNA, leading to atherosclerosis, carcinogenesis, cardiovascular diseases, cellular aging, chronic inflammation, diabetes, hypertension, mutagenesis, neurodegenerations, rheumatoid arthritis, stroke, septic shock, and other degenerative diseases [7,14,15,16,17]. Antioxidants prevent oxidation of proteins and generation of ROS and RNS that may reduce free-radical damage to normal tissues by combating oxidative stress [17,18,19].

The skin produces a dark pigment known as melanin. The production of melanin prevents the skin from UV-induced damage [9]. However, overproduction and accumulation of melanin cause cutaneous hyperpigmentation, leading to esthetic complications such as melasma, freckles, senile lentigines, nevus, and ephelis [9,20]. Tyrosinase is a glycoprotein found in the membranes of melanosomes that are responsible for melanin biosynthesis by hydroxylation of l-tyrosinase to 3,4-dihydroxyphenylalanine (l-DOPA) and subsequent oxidation of l-DOPA to dopaquinone [21]. Due to the spontaneous polymerization, dopaquinone is finally converted to melanin [22]. Over-produced melanin should be controlled to maintain the skin integrity. That is why the discovery of novel tyrosinase inhibitors for the development of cosmeceutical formulations has received a significant interest [23,24,25].

Antimicrobial preservatives are added to cosmetic products to maintain the microbiological purity during the entire period of its applications [26]. Instead of using cosmetics preservatives such as methylparabens, natural antimicrobial compounds having other functions are better and more beneficial for human health [27,28,29]. The secondary metabolites isolated from bacterial resources may have both preservative and antimicrobial properties that inhibit the colonization of bacterial pathogens (*Propionibacterium acnes*, *Staphylococcus epidermidis*, and *Staphylococcus aureus*) in the skin [9,26,28].

Bioactive compounds produced by soil or marine bacteria, especially actinobacteria, are unexploited and still unexplored [23,30]. Various bioactive compounds that are applicable in the cosmeceutical and cosmetic industry, including those with anti-aging, antioxidant, anti-tyrosinase, antimicrobial, and non-cytotoxic properties, have great potential for novel uses.

There are numerous studies on bioactive materials isolated from plants that are currently used in cosmeceutical formulations, but very few studies on bioactive materials from bacteria are available [9,21,24,25,26,27,31]. This study aimed to evaluate the cosmeceutical potential of ethyl acetate extracts from the bacterial strain *Streptomyces* sp. T65 for antioxidant, anti-aging, anti-tyrosinase, and antibacterial activities and any cytotoxic effects on different mouse and human cell lines. In addition, we aimed to synthesize mesoporous silica particles. Finally, the main goal of this study was to develop the final cosmetic product for topical application using bioactive material extracted from soil microorganisms.

## 2. Materials and Methods

### 2.1. Reagents, Cell Lines, and Equipment

All solvents used were of analytical grade. B16-F10 melanoma cell line, B16-F1 mouse melanoma cell line, and human keratinocyte cell line (HaCaT) were purchased from American Type Culture Collection (ATCC, Manassas, VA, USA). Dulbecco’s modified Eagles’ medium (DMEM), penicillin–streptomycin, and heat-inactivated fetal bovine serum (HI FBS) were purchased from Gibco (Thermo Fisher Scientific Korea Ltd., Seoul, South Korea). The Cell Counting Kit-8 (CCK-8) was purchased from Dojindo (Kumamoto, Japan). A mouse macrophage cell line RAW264.7 and CCD-986Sk human fibroblasts were purchased from the Korean Cell Line Bank (Seoul, South Korea). Lipopolysaccharide (LPS, *Escherichia coli*, serotype O11:B4), sulfanilamide, naphthylethylenediamine dihydrochloride, mushroom tyrosinase, porcine pancreatic elastase, l-tyrosine, ascorbic acid, arbutin, *N*-Succ-(Ala)_3_-*p*-nitroanilide, 3-(4,5-dimethylthiazol-2-yl)-2,5-diphenyl tetrazolium bromide (MTT), collagen type I, Tween 20, tetramethylbenzidine (TMB), tetraethyl ortho-silicate (TEOS), pluronic P-123 (poly(ethylene glycol)-block-poly(propylene glycol)-block-poly(ethylene glycol); PEG-PPG-PEG), and *α*-melanocyte-stimulating hormone (*α*-MSH) were purchased from Sigma-Aldrich (St. Louis, MO, USA). 1,1-Diphenyl-2-picrylhydrazyl (DPPH) and oleanolic acid were purchased from Aldrich (St. Louis, MO, USA). COL1A1 (Collagen, type I, alpha 1) primary antibody and secondary antibody conjugated with HRP (Horseradish peroxidase) were purchased from Santa Cruz Biotechnology (Santa Cruz, CA, USA). A SpectraMax 340PC384 Microplate Reader (Molecular Devices, Sunnyvale, CA, USA) was used for 96-well plate reading.

### 2.2. Pathogenic Bacterial Strains

*Staphylococcus epidermidis* KACC 13234 and *Pseudomonas aeruginosa* KACC 10185 were purchased from the Korean Agriculture Culture Collection (KACC, Jeonju, South Korea); *Bacillus subtilis* KEMB 51201-001, *Escherichia coli* KEMB 212-234, and *Staphylococcus aureus* KEMB 7301-069 were obtained from the Korea Environmental Microorganisms Bank (KEMB, Suwon South Korea); and *Propionibacterium acnes* KCTC 3314 was purchased from the Korean Collection for Type Cultures (KCTC, Jeongeup, South Korea).

### 2.3. Isolation and Preservation

Different soil samples were collected from reclaimed grasslands in Hwaseong (37°16′10″ N 126°45′43″ E), and Kyonggi University forest (37°18′1″ N 127°2′20″ E) in Korea. Bacteria were isolated using a method previously described [9]. Colonies were streaked on R2A plates every 1 week for short-term preservation and stored at −80 °C as a suspension in R2A broth supplemented with 20% (*v*/*v*) glycerol for long-term preservation.

### 2.4. Screening, Identification, and Phylogenetic Position of Isolated Strains

Screening for cosmetics and antimicrobial activities of isolated strains were completed as previously described [9]. Bacteria having functions were identified using 16S rRNA gene sequencing. Genomic DNA of strains was extracted using an InstaGene Matrix kit (Bio-Rad, Hercules, CA, USA), and the 16S rRNA gene was amplified by PCR using the universal bacterial primer 27F and 1492R [32]. The PCR product was purified with a multiscreen-filter plate (Millipore Corp., Bedford, MA, USA), and was sequenced with an Applied Biosystems 3770XL DNA analyzer using a BigDye Terminator cycle sequencing kit v.3.1 (Applied Biosystems, Foster City, CA, USA). A nearly complete sequence was complied with SeqMan software (DNASTAR Inc., Madison, WI, USA). The closest strain of isolated functional strains was identified using EzBioCloud [33] and the NCBI GenBank database [34]. Related 16S rRNA sequences were obtained from GenBank, and phylogenetic analysis was accomplished using MEGA7 [35].

### 2.5. Culture of Bacteria

*P. acnes* was cultured by incubation at 37 °C for 3–4 days anaerobically in Schaedler anaerobe broth (Oxoid). For the anaerobic culture, BBL anaerobic jar with GasPak EZ Gas Generating Container System (Becton Dickinson, NJ, USA) was used. *S. epidermidis* and *S. aureus* were cultured in TSB (Tryptic soy broth) medium (Oxoid) at 37 °C for 24 h aerobically. *E. coli*, *P. aeruginosa*, and *B. subtilis* were cultured in LB (Luria-Bertani) (Oxoid) medium. Isolated bacterial strains were cultured in R2A at 28 °C for 4–5 days.

### 2.6. Fermentation

For the fermentation process, the inoculum was prepared in R2A broth at 28 °C for 4–5 days at 150 rpm. Strain T65 was fermented using 1–2% inoculum in ISP2 (International *Streptomyces* Project 2) medium at 28 °C for 1 week at 140 rpm.

### 2.7. Extraction

The harvested culture broth was centrifuged at 11,305× *g* for 20 min at 4 °C with large capacity centrifuge 1736R (LABOGENE, Seoul, Korea). The culture supernatant was filtered with 150 mm size filter paper (Whatman 1001-150, GE Healthcare, Maidstone, UK) to eliminate cell debris and concentrated with a rotary evaporator at 40 °C. The concentrated crude product was then extracted two times with equal volume using five different solvents (*n*-hexane, di-ethyl ether, di-chloromethane, chloroform, and ethyl acetate). Ethyl acetate was found to be the best solvent, and further analyses were conducted using ethyl acetate extract. The organic layer was separated by a separation funnel and evaporated to dryness. Finally, the dried crude product was dissolved in methanol for further assessment.

### 2.8. Collection of Active Fractions by Preparative HPLC (Prep-HPLC)

Active fractions of crude culture extract of T65 were collected using preparative HPLC (Agilent 1200 series). Shim-pack-PREP-ODS (K) C18 reverse column (30 mm i.d. × 25 cm) with a 15 μm particle size was used as the stationary phase. For the mobile phase, 0.1% HCHOOH water (solvent A) and acetonitrile (solvent B) were used. Solvent B concentration was 10% to 100% from 0 min to 60 min and 100% from 60 min to 75 min were used. The flow rate was 15 mL/min. Multi-wave detector (MWD) was used with 208, 230, 254, and 280 nm. Collected fractions from 14 min to 21 min were evaporated to dryness and dissolved in methanol (T65 crude product) for further assessments.

### 2.9. Cell Viability of HaCaT Cell

Cell viability of HaCaT cells was determined with a CCK-8 (Cell Counting Kit-8) assay according to the manufacturer’s instructions. The HaCaT cells were seeded in 96-well plates at 10^4^ cells per well and then incubated at 37 °C for 24 h in Dulbecco’s modified Eagles’ medium (DMEM) containing 10% fetal bovine serum (FBS). Cells were treated with different concentrations of T65 crude product (10 mg/mL, 1 mg/mL, 100 μg/mL, 10 μg/mL, 1 μg/mL, 100 ng/mL, and 1 ng/mL), and incubated at room temperature for 2 h in a humidified atmosphere containing 5% CO_2_ with the addition of 10 μL of CCK-8 reagent. The absorbance of the reaction mixture was measured with a microplate spectrophotometer at 450 nm and the percentage of cell viability was calculated.

### 2.10. Evaluation of Antioxidant Activities

#### 2.10.1. Evaluation of Toxicity in RAW264.7 Cells

RAW264.7 cells were maintained in DMEM containing 10% FBS, 100 U/mL of penicillin, and 100 μg/mL of streptomycin at 37 °C in a 5% CO_2_ incubator. RAW264.7 cells were seeded in a 96-well flat-bottom microtiter plate at a density of 10^4^ cells per well with different concentrations (0–1 mg/mL) of T65 crude product and incubated at 37 °C for 24 h in a 5% CO_2_ incubator. After 24 h incubation, cells were washed twice with phosphate-buffered saline (PBS), and 190 μL of fresh medium and 10 μL of MTT working solution (5 mg/mL) were added to each well, and the plate was then incubated at 37 °C for 4 h in a 5% CO_2_ incubator. Then, the supernatant was removed, and the formazan crystals that formed were solubilized by adding 150 μL of DMSO (dimethyl sulfoxide) in each well for 10 min at 37 °C in a 5% CO_2_ incubator. The intensity of the dissolved formazan crystals was quantified using a microplate reader at 570 nm.

#### 2.10.2. Nitric Oxide Determination

The RAW264.7 cells (10^5^ cells/mL) were pretreated with various concentrations of T65 crude product (15.5–125 μg/mL) for 30 min, followed by stimulation with 1 μg/mL LPS for 24 h. The concentration of NO released from the cells was determined by Griess reagent using the standard curve of NO_2_^–^ [36]. One-hundred microliters of culture supernatant was incubated with 100 μL of Griess reagent (mixture of *N*–1–naphthylethylenediamine dihydrochloride (NEDHC) and sulfanilamide) at room temperature for 20 min in the dark. The absorbance was measured at 540 nm.

#### 2.10.3. DPPH Free Radical Scavenging Assay

DPPH free radical scavenging activities were conducted according to a previously described method [9]. The crude product of T65 culture extract was diluted to concentrations 600, 200, 100, 20, and 4 μg/mL in methanol. The reaction mixture of 180 μL was made with 90 μL of 0.1 mM DPPH (dissolved in MeOH) and 90 μL of sample solutions of different concentrations (Table S1). Test reactions were mixed thoroughly in 96-well plates, incubated at 37 °C for 30 min, and absorption was measured at 516 nm with a spectrophotometer. The percentage of DPPH inhibition was calculated as follows:

(1) $$\mathrm{Inhibition}(\%)=[1-\frac{\left(\mathrm{ODexp}-\mathrm{ODcon}\right)}{\left(\mathrm{ODstd}-\mathrm{ODbln}\right)}]\times 100$$

where ODexp is absorbance of the experimental sample; ODcon is absorbance of the control; ODstd, the absorbance of the standard; and ODbln, the absorbance of the blank.

### 2.11. Evaluation of Anti-Aging Activities

#### 2.11.1. Evaluation of Cytotoxicity in CCD-986Sk Cells

Cell cytotoxicity of various concentrations (0–1 mg/mL) of T65 crude product in human skin fibroblast cells (CCD-986Sk) was determined by MTT assay as previously described for MTT assay of RAW264.7 cells.

#### 2.11.2. Human Fibroblast Proliferation Using CCD-986Sk Cells

Human dermal fibroblast (HDF) cell proliferation was determined in CCD-986Sk cells using a CCK-8 assay. CCD-986Sk cells were seeded in 96-well plates at 5 × 10^3^ cells/well and then incubated at 37 °C for 24 h in DMEM containing 10% FBS. Cells were treated with different concentrations of T65 crude product (0–1 mg/mL) and incubated at room temperature for 2 h in a humidified atmosphere containing 5% CO_2_ with the addition of 10 μL of CCK-8 reagent. The absorbance of the reaction mixture was measured with a microplate reader at 450 nm and the percentage of viable cells was determined in comparison to the absorbance of the untreated cells.

#### 2.11.3. Collagen Type I Synthesis Assay

Collagen type I synthesis was assayed by an enzyme-linked immunosorbent assay (ELISA). CCD-986Sk cells were seeded in 96-well plates at a density of 5 × 10^3^ cells/well in DMEM containing 10% FBS and incubated at 37 °C for 24 h. Various concentrations of T65 crude product (31.25–250 pg/mL) was added in FBS-free medium for 24 h. Then, 100 μL of culture medium and collagen type I was added into collagen-coated 96-well plates and incubated at 37 °C for 24, 48, and 72 h. Each well was washed with 0.05% phosphate-buffered saline with 0.1% Tween 20 (PBST) and COL1A1 primary antibody was added and incubated for 1 h. Again, the wells were washed with 0.05% PBST and a secondary antibody conjugated with HRP (horseradish peroxidase) was added and incubated for 1 h. Each well was washed with 0.05% PBST, and TMB (tetramethylbenzidine) was added. After obtaining the desired color intensity (blue), the reaction was terminated by adding 0.5N H_2_SO_4_, which turned the color of the solution yellow. The absorbance was measured at 450 nm with a microplate reader, and collagen type I production was determined.

#### 2.11.4. Elastase Inhibition Assay

Porcine pancreatic elastase (PPE) inhibition activities were assayed according to a procedure previously described [9]. The T65 crude product was diluted to concentrations of 3000, 1000, 500, and 100 μg/mL in methanol. The reaction mixture was prepared with 0.2 M Tris-HCl buffer (pH 8.0), 0.5 mM N-Succ-(Ala)_3_-ρ-nitroanilide (SANA) as a substrate, porcine pancreatic elastase (3.5 U/mL in 0.2 M Tris-HCl buffer; pH 8.0), and the inhibitor (sample). Each sample was pre-incubated at 37 °C for 15 min, and the total reaction mixture was incubated at 37 °C for 20 min. The absorption was measured at 400 nm. The total reaction mixture of 150 μL was prepared as shown in Table S2. The final concentrations of the sample in the reaction mixture were 300, 100, 50, and 10 μg/mL. The percentage of PPE inhibition was calculated using Formula (1).

### 2.12. Evaluation of Whitening Activities

#### 2.12.1. Evaluation of Cytotoxicity in B16F1 Cells

Cytotoxicity of T65 crude product to B16F1 melanoma cells was determined by an MTT assay. B16F1 cells were seeded in 96-well plates at 10^4^ cells per well and then incubated at 37 °C for 24 h in DMEM containing 10% FBS. Cells were treated with different concentrations of T65 crude product (0–1 mg/mL) and incubated at room temperature for 2 h in a humidified atmosphere containing 5% CO_2_. The absorbance of the reaction mixture was measured with a microplate spectrophotometer at 450 nm and the percentage of cell viability was calculated.

#### 2.12.2. Melanin Synthesis Inhibition in B16F10 Cells

B16F10 cells were pretreated with *α*-MSH in 6-well plates at 10^5^ cells per well and incubated at 37 °C for 24 h in DMEM containing 10% FBS to promote melanin synthesis. The cells were incubated with various concentrations of T65 crude product (31.25–125 μg/mL) and 100 μg/mL of arbutin as a positive control in the presence or absence of *α*-MSH for 48 h. The melanin synthesis inhibition in B16F10 melanoma cells was observed.

#### 2.12.3. Mushroom Tyrosinase Inhibition Assay

Mushroom tyrosinase inhibition activities were determined as previously described [9]. The T65 crude product was diluted to concentrations of 3000, 1000, 500, and 100 μg/mL in methanol. The reaction mixture was prepared with 0.1 M potassium phosphate buffer (pH 6.8), 3 mM l-tyrosine solution [dissolved in DW (distilled water)], and 2000 U/mL mushroom tyrosinase (dissolved in 0.05 M potassium phosphate buffer, pH 6.8; Sigma) in 96-well plates. Total test mixture of 150 μL (120 μL phosphate buffer, 10 μL l-tyrosine, 15 μL sample solution, and 5 μL mushroom tyrosinase; Table S3) was incubated at 37 °C for 10 min and absorption was measured at 475 nm. The final concentrations of the sample in the reaction mixture were 300, 100, 50, and 10 μg/mL. The standard was without the sample solution, the control was without l-tyrosine, and the blank was without l-tyrosine and the sample solution. The percentage of tyrosinase inhibition was calculated by applying Formula (1).

### 2.13. Analysis of Amino Acids

The free amino acid of T65 crude product was determined by the GC-FID (gas chromatography–flame ionization detector) method at the Korea Polymer Testing and Research Institute (Koptri). For the GC-FID method, the machine used was an Agilent 6890N GC-FID; column, ZB-AAA (10 m × 0.25 mm); injection part temperature, 250 °C; injection column, 2 μL; split ratio, 5:1; temperature condition, 110 °C → 32 °C/min → 320 °C; detector, FID @ 320 °C; carrier, nitrogen gas, 1.5 mL/min; maker, Phenomenex; amino acid standard; and concentration, 200 μmol/L.

The composite amino acids of T65 crude product was determined by the GC-FID (gas chromatography–flame ionization detector) method at the Korea Polymer Testing and Research Institute (Koptri). For GC-FID, the machine used was an Agilent 6890N GC-FID; column, ZB-AAA (10 m × 0.25 mm); injection part temperature, 250 °C; injection column, 2 μL; split ratio, 5:1; temperature condition, 110 °C → 32 °C/min → 320 °C; detector, FID @ 320 °C; carrier, nitrogen gas, 1.5 mL/min; maker, Phenomenex; amino acid standard; and concentration, 200 μmol/L.

### 2.14. Fatty Acid

Fatty acid of T65 crude product was determined by the GC-FID (gas chromatography–flame ionization detector) method at the Korea Polymer Testing and Research Institute (Koptri). For GC-FID, the machine used was Agilent 6890N GC-FID; column, Supelco SP–2500 (100 m × 0.25 mm × 0.20 μm); injection part temperature, 250 °C; injection column, 1 μL; split ratio, 50:1; temperature condition, 100 °C (4 min) → 3 °C/min → 240 °C (15 min); detector, FID @ 285 °C; carrier, nitrogen gas, 0.8 mL/min; maker, SUPELCO 37 Component FAME Mix; and concentration, 0.5 mg/mL.

### 2.15. Antimicrobial Activities

Inhibition of pathogenic bacteria was performed by the disc diffusion method. One-hundred microliters of *P. acnes* culture at 10^8^ CFU (colony-forming unit)/mL was spread and incubated at 35 °C anaerobically for 2–3 days on Schaedler agar plates along with a 6 mm disc (Whatman) containing 15 μg of crude extract dissolved in methanol, and the zones of inhibition were measured. Similarly, 100 μL of *S. epidermidis*, *S. aureus*, *B. subtilis*, *E. coli*, and *P. aeruginosa* at 10^8^ CFU/mL were spread and incubated at 35 °C aerobically on R2A or LBA plates for 1-2 days and the zones of inhibition were measured.

### 2.16. Synthesis of Mesoporous Silica Particle

The structure-inducing polymer was dissolved in deionized water to prepare a micelle solution. Mesoporous silica materials were synthesized according to the methods described in the literature [37]. For the synthesis of mesoporous silica particles (SBA-15), 10 g of Pluronic P123 (EO_20_PO_70_EO_20_, BASF Corporation, Florham Park, NJ, USA), was dissolved in 55 mL of 2 M HCl, followed by stirring at room temperature for 30 min. Furthermore, 22 g of tetraethylorthosilicate (TEOS) was added to the solution and further stirred for 30 min and placed at 36 °C for 24 h and then put into the oven, in which temperature was maintained at 100 °C, and was left for 4 days under the static condition. After 4 days, the solution changed to a cloudy solution in the bottle. The cloudy solution was filtered with the two layers of cellulose filter paper and washed with EtOH (two times) and DI (deionized) water (two times). The resulting filtrated powder was dried in the 120 °C convection oven and put into the muffle furnace (Hanyang Scientific Equipment Co., Ltd., Seoul, South Korea). Six samples were synthesized with the same conditions but in a different batch. Transmission electron micrographs (TEM) of synthesized SBA-15 were taken in Seoul National University by transmission electron microscopy (Talos L120C; FEI).

### 2.17. BET Surface Area Analysis

The particle size of silica was measured by the Mastersizer 2000 (Malvern Instruments Lt., Malvern, United Kingdom). For the sample preparation of BET (Brunauer–Emmett–Teller) analysis, 5 g of P-123 was added to 76 g of 2M HCl and stirred at 250 rpm for 24 h. After 24 h, when P-123 was completely dissolved, 160 mL of DI (deionized) water and TEOS were uniformly (15 mL/min) added and stirred at 750 rpm for 24 h. After that, the formed powder was separated by filtering and the separated powder was placed into a thimble and washed with Soxhlet for 24 h. Then, it was washed with DI water and burned in a muffle furnace (Hanyang Scientific Equipment Co., Ltd., South Korea). BET surface area analyses were conducted to confirm the performance of the powder produced by SBA-15 synthesis. BET analyses were completed by the Inha University and Changwon National University.

The gas sorption analyser (Autosorb iQ, Quantachrome Instruments, Ashland, OR, USA) was employed to examine the surface area and pore size distributions of prepared mesoporous silica materials. The surface area and pore size distributions were calculated using ASiQwin software (Anton Paar Quanta Tec Inc., Boynton Beach, FL, USA) based on adsorption–desorption isotherms. The pristine synthesized particles were degassed at 300 °C/3h, then N_2_ adsorption and desorption isotherms were measured at a temperature of −196 °C. Multipoint BET analysis was applied for the total surface area calculation.

### 2.18. Sustainability Assessment

To confirm the presence of T65 in embedded SBA-15, the following performance was conducted as follows: 12 g of SBA-15 and 1.2 g of T65 crude product were mixed in 500 mL acetonitrile. The solution was stirred for 18 h under 30 °C. After filtration, of the mixture was filtered and filtered particles were dried in a 100 °C convection oven. To find the T65 particle itself in SBA-15, 1 g of dried T65 embedded in SBA-15 was dissolved in acetonitrile and 25 mL of 3 M fluoric acid was added. The mixture was stirred at room temperature for 24 h until the solution became clear. Then, the solution was filtered, and the filtrate solution was used as a sample. Samples obtained from the above experiments were analyzed by HPLC.

To determine the property of controlled release, 35 mL of embedded SBA-15 was poured into 50 mL of mineral oil (M3516; Sigma-Aldrich, St. Louis, MO, USA) and mixed well. Then, 50 mL of DI water was added to the mixture and shaken under room temperature for 6 h, and then left on the desk for 12 h. When the liquid layer was separated, the water layer was discarded, and 1 mL of oil layer was collected as a sample for HPLC analysis. The same experiment was repeated on the second and third day and the sample was again analyzed by using HPLC.

### 2.19. Cosmetic Formulation and Application

#### 2.19.1. Formulation and Stability Test

A cosmetic product was formulated from an emulsifier-type cream. Cosmetic formulation stability was determined by keeping the cosmetic product at various temperatures (37, 45, and 60 °C) including in the freezer, refrigerator, and at room temperature for up to 28 days. The stability of cosmetic cream was observed in the first, second, third, and fourth week.

#### 2.19.2. Clinical Trials, Volunteers, and Application Methods

Functional cosmetic cream containing T65 crude product was applied to 21 female volunteers for determination of wrinkle improvement and whitening efficacy through skin roughness and skin brightness. All the experimental procedure for human involvement for this study were approved by the Ellead Institutional Review Board (IRB) (EL-P-7400). Informed consent was obtained from all individual participants. Tests were conducted before the application of the product, after 2 weeks and after 4 weeks. Approximately 5 mg of cosmetic product was applied two times a day (morning and evening) on the face after cleansing the face, and it was softly spread according to the skin texture. The volunteers were not allowed to use any other creams or products 1 week prior to the trial and during the trial.

#### 2.19.3. Image Capturing and Processing Methods

Pictures were captured by VISIA-CR. The roughness of images was analyzed by PRIMOS software (PRIMOS version 5.8E, Canfield Scientific, Inc., Parsippany-Troy Hills, NJ, USA). Average roughness (Ra) and maximum roughness depth (Rmax) were calculated using the following formula:

Ra or Rmax rate (%) = (value before treatment − value after treatment)/value before treatment × 100(2)

The brightness of images was analyzed by Chromameter CR-400. L* is the brightness parameter and is measured by the following formula:

L* increasing rate (%) = (value before treatment − value after treatment)/value before treatment × 100(3)

### 2.20. Statistical Analysis

Statistical calculations of the IC_50_ value were performed by OriginPro 8.5 statistical software (OriginLab Corporation, Northampton, MA, USA). All experiments were conducted in triplicate, and all results were presented as mean ± SD of three separate experiments. Data were analyzed by independent sample *t*-test one-way analysis of variance (one-way ANOVA), followed by Tukey’s post-hoc test using OriginPro 8.5. Differences were considered significant at *p* < 0.05.

## 3. Results and Discussions

### 3.1. Isolation, Selection, and Phylogeny of Selected Active Strains

A total of 2285 strains of bacteria were isolated from the collected soil samples. Screening results showed 102 strains of bacteria had functions applicable to the cosmeceuticals (Table S4). On the basis of primary screening, *Streptomyces* sp. T65 was found to have higher activities for all cosmetic applications, namely, anti-oxidant, anti-elastase, anti-tyrosinase, and antimicrobial activities. Finally, strain T65 was chosen for further assessment to identify its potential application in cosmeceuticals. Phylogenetic analysis showed that strain T65 formed a clade with *Streptomyces bungoensis* DSM 41781^T^ (the closest strain on the basis of 16S rRNA gene sequence) with a strong bootstrap value (Figure S1).

### 3.2. Detection and Collection of Bioactive Material

Mixed bioactive materials from T65 ethyl acetate culture extract was collected from Prep-HPLC. Bioactive materials were measured at 208, 230, 254, and 280 nm by detectors. The fractions were collected on the basis of time (from 14 min to 21 min). The collected fractions were evaporated to dryness and dissolved in methanol (T65 crude product) and were used for all the assessments, including antimicrobial activities.

### 3.3. Cytotoxicity in HaCaT Cell

Cell viability in the human keratinocyte cell line (HaCaT cell) remained unaffected by T65 crude product up to 10 mg/mL. On the other hand, cell viability increased in a dose-dependent manner when concentrations of 10 ng/mL to 10 mg/mL of the T65 crude product were applied (Figure S2). This concentration range resulted in more than 100% cell viability, indicating that it promoted the proliferation of HaCaT cells and was, thus, non-toxic to the HaCaT cell line.

### 3.4. Antioxidant Activities

#### 3.4.1. Cytotoxicity in RAW264.7 Cell

**Figure 1:**
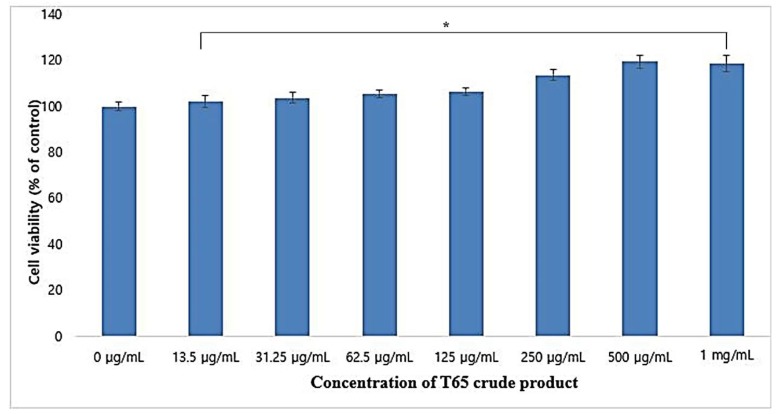
Assessment of cytotoxicity of T65 crude product in RAW264.7 cells. Values are given as mean ± SEM (standard error of the mean). * *p* < 0.05 compared to control.

Cytotoxicity of the T65 crude product was assessed by cell viability in RAW264.7 macrophages by the MTT assay method. T65 crude product showed no cytotoxic effect on RAW264.7 cell lines (Figure 1).

On the contrary, T65 crude product helped to proliferate RAW264.7 cells in a dose-dependent manner when treated with 13.5 to 1000 μg/mL. When 1000 μg/mL T65 crude product was used, the cell viability of RAW264.7 cells were 118.7% (*p* < 0.05). These results showed that the T65 crude compound was non-toxic up to 1 mg/mL and could be used in cosmeceutical formulations.

#### 3.4.2. Inhibition of Nitric Oxide (NO) Production

**Figure 2:**
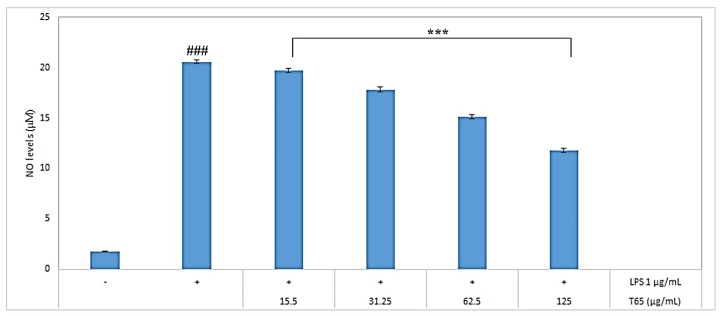
Evaluation of NO inhibition by T65 crude product. Values are given as mean ± SEM. ### *p* < 0.001 compared to untreated control and *** *p* < 0.001 compared to lipopolysaccharide (LPS) alone.

Griess reagent was used to determine the NO levels. To determine the NO inhibition, RAW264.7 cells were treated with 1 μg/mL LPS (lipopolysaccharide) along with various concentrations of T65 crude product. RAW264.7 cells were activated by LPS, and NO production was measured as nitrite concentration in the culture medium. Compared to LPS alone, T65 crude product significantly (*p* < 0.001) inhibited the nitrite concentration in the LPS-stimulated RAW264.7 cells in a concentration-dependent manner when used from 15.5 to 125 μg/mL (Figure 2). The macrophages were LPS-Toll-like expression of inducible NO synthase (iNOs) by signaling cell activation via receptors [38]. LPS is an activator of macrophages and plays an important role in the production of NO in mammalian cells [39]. NO is a free radical produced by immunocompetent cells such as macrophages [40]. Production of NO has a phagocytic effect on macrophages. Thus, macrophage cell line RAW264.7 was selected for antioxidant assay. For a long time, considerable attention has been given to the quest of natural antioxidants to inhibit NO production [41]. At 125 μg/mL, T65 crude product sufficiently decreased the NO levels by 57.4% compared to the NO produced by 1 μg/mL LPS. On the basis of inhibition of NO produced in LPS-induced RAW264.7 macrophage cell line, the T65 culture extract could be considered a potent antioxidant.

#### 3.4.3. DPPH Radical Scavenging Activity

**Figure 3:**
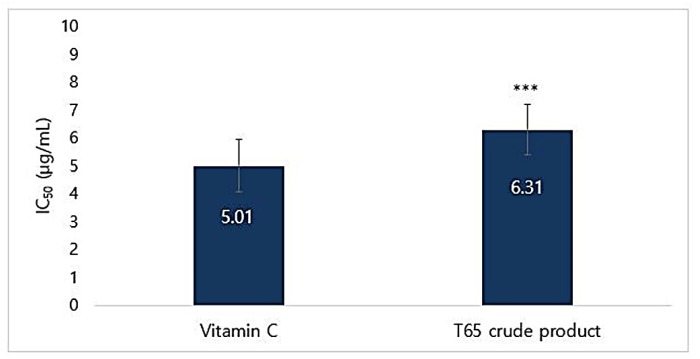
IC_50_ of 1,1-diphenyl-2-picrylhydrazyl (DPPH) radical scavenging activities of T65 crude product and vitamin C. Data are given as mean ± SEM. Statistical significance is shown by ANOVA (*** *p* < 0.001).

DPPH radical scavenging percentages at different concentrations and IC_50_ value of T65 crude product are given in Table S5. Ascorbic acid (vitamin C) was used as a standard because it is used in lotions, creams, serums, and patches, which are well known to have powerful antioxidant activities for topical applications [42]. IC_50_ for DPPH radical scavenging activities for vitamin C and T65 secondary product were found to be 5.01 and 6.31 μg/mL, respectively (Figure 3).

There are only a few studies about the antioxidant activities from bacterial isolates compared to those from plant materials and most studies have focused on well-established plant products [43,44,45,46,47,48,49]. A concentration of 10 μg/mL product was able to scavenge 68.66 ± 2.83% of DPPH-free radical. The IC_50_ of T65 crude product was almost comparable to that of the strongest antioxidant vitamin C, which showed that it could be used as an antioxidant agent in the formulation of cosmeceuticals.

### 3.5. Antiaging Activities

#### 3.5.1. Cytotoxicity in CCD-986Sk Cells

**Figure 4:**
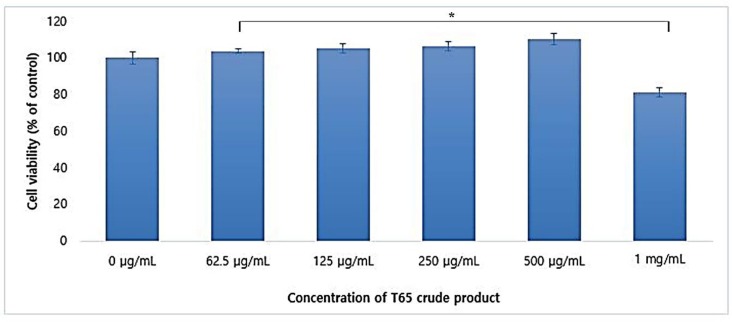
Cell viability assessment of T65 crude product in human dermal fibroblast (HDF) cells using CCD-986Sk cell line. Data are given as mean ± SEM. * *p* < 0.05 compared to control.

Cytotoxicity of crude compounds of T65 in human dermal fibroblast (HDF) cells was evaluated in the CCD-986Sk cell line using the MTT assay. When the concentration of T65 crude product from 0 to 1 mg/mL were supplied, the HDF cells proliferated in a dose-dependent manner until the concentration of 500 μg/mL. This result showed that the crude compounds from T65 were non-cytotoxic to HDF cells. However, a concentration of more than 1 mg/mL was found to be cytotoxic, and only 81.34% of the cells survived compared to the control (Figure 4).

#### 3.5.2. Proliferation of HDF

**Figure 5:**
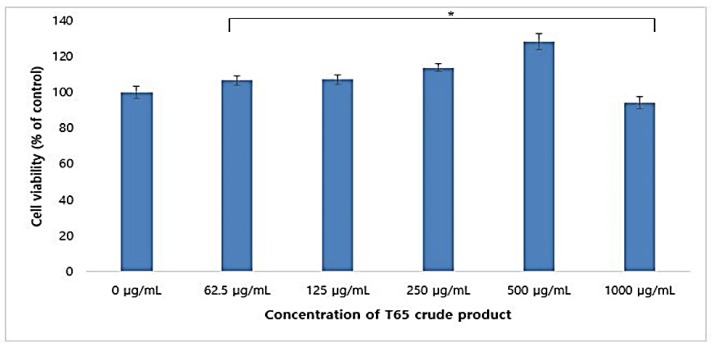
Proliferation of human dermal fibroblast (HDF) cells evaluated in CCD-986Sk cell line. Data are given as mean ± SEM. * *p* < 0.05 compared to control.

The proliferation of HDF cells was determined using the CCD-986Sk cell line. T65 crude product proliferated the HDF cells in a concentration-dependent manner from 0 to 500 μg/mL (Figure 5). However, it was cytotoxic at a concentration of 1 mg/mL. Human dermal epithelial cell growth confirmed wrinkle improvement ability through the change in the primary cultured cells isolated from human dermal cells and secreted collagen [50,51].

#### 3.5.3. Collagen Type I Synthesis

**Figure 6:**
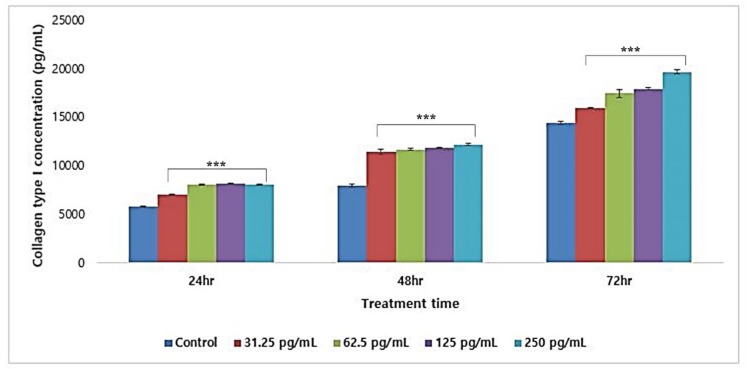
Synthesis of collagen type I using T65 crude product in 24, 48, and 72 h. Data are given as mean ± SEM. ***p < 0.05 compared to control in respective hours.

Synthesis of collagen type I was detected by ELISA. The crude product of T65 at 250 pg/mL increased the concentration of collagen type I in a dose-dependent manner up to 145.91% ± 9.11% (mean ± SD; mean of 24, 48, and 72 h). The result of three experiments at 24, 48, and 72 h showed the synthesis of type I collagen (Figure 6). Thus, dermal cell secretion of collagen accompanied by human dermal epithelial cell growth confirmed the wrinkle-improvement ability of T65 compounds.

#### 3.5.4. Elastase Inhibition Assay

**Figure 7:**
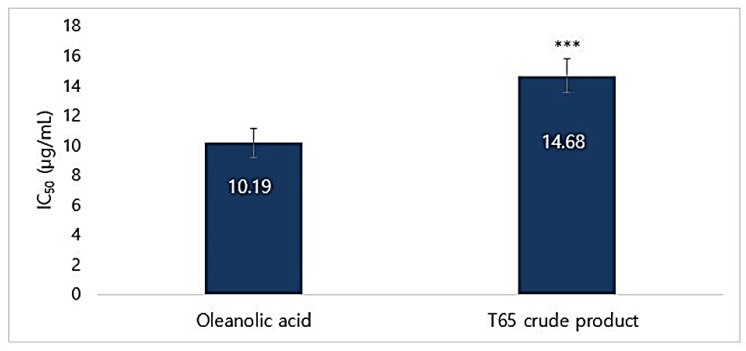
IC_50_ of porcine pancreatic elastase (PPE) inhibition using T65 crude product and oleanolic acid. Data are given as mean ± SEM. Statistical significance is shown by ANOVA (*** *p* < 0.001).

The T65 crude product showed strong porcine pancreatic elastase inhibition activity. The IC_50_ values of T65 and oleanolic acid (commercialized inhibitor used in cosmeceuticals) were 10.19 and 14.68 μg/mL, respectively (Figure 7). T65 showed a slightly lower inhibitory activity in comparison to the positive standard. However, T65 crude product inhibited 84.98% of elastase at a concentration of 300 μg/mL (Table S6). These results showed that the compounds of T65 from the ethyl acetate extract had a strong potential to be used in cosmetic formulations.

### 3.6. Whitening Activities

#### 3.6.1. Cytotoxicity in B16F1 Cells

**Figure 8:**
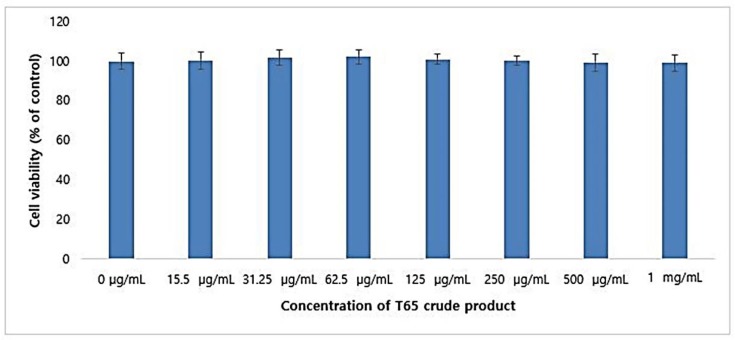
Cytotoxicity of T65 crude product in B16F1 melanoma cells. Data are given as mean ± SEM.

The result of cytotoxicity of T65 crude product on B16F1 melanoma cells by the MTT assay is shown in Figure 8. The outcome of incubation of T65 crude product in B16F1 cells showed non-toxic effects up to 1 mg/mL concentration. B16F1 cells proliferated in a dose-dependent manner up to a concentration of 62.5 μg/mL, but a significant (*p* < 0.05) dose-dependent decrease in cell viability was evaluated from 125 to 1000 μg/mL. However, at a concentration of 1 mg/mL, 99.12% ± 4.16% of viable cells were observed (Figure 8). The experimental results showed that the ethyl acetate extract of strain T65 culture was non-cytotoxic to the B16F1 melanoma cell line and could be used in the formulation of cosmetic products for whitening of human skin.

#### 3.6.2. Melanin Synthesis Inhibition in B16F10 Cells

**Figure 9:**
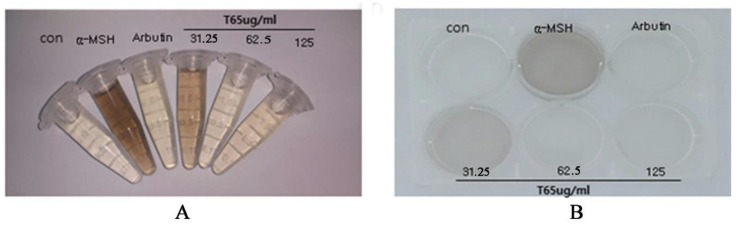
Confirmation of whitening effect in B16F10 cells by T65 crude product. *α*-MSH: *α*-melanocyte stimulating hormone used to stimulate melanin. (**A**) Experiment performed in Eppendorf’s tube; (**B**) experiment performed in 6-well plate.

For confirmation of the whitening effect, B16F10 cells, a cell line that secretes and produces melanin in cells, were used. To promote melanin synthesis, B16F10 was supplemented with 1 μg of *α*-MSH. Arbutin (100 µg/mL; a commercialized whitening agent) was used as the positive control. The microscopic observation of this experiment showed almost complete inhibition of melanin by application of 125 µg/mL of T65 culture extract in 48 h (Figure 9A, B). This experiment proved that T65 culture extract has the potential to inhibit melanin synthesis in a dose-dependent manner and can be used as a whitening agent for the formulation of cosmetic products for topical applications.

#### 3.6.3. Mushroom Tyrosinase Inhibition Assay

**Figure 10:**
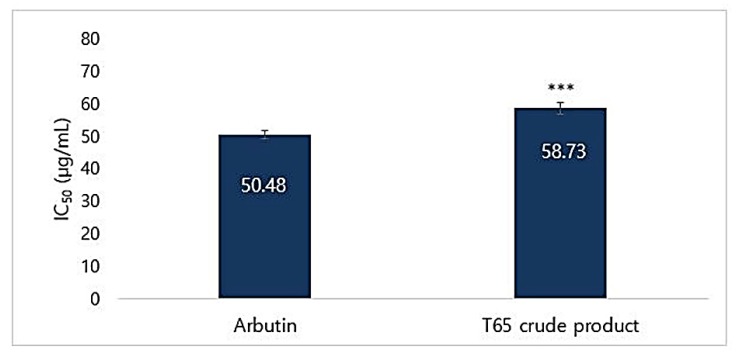
IC_50_ of mushroom tyrosinase inhibition by T65 crude product and arbutin. Data are given as mean ± SEM and statistical significance is shown by ANOVA (*** *p* < 0.001).

The T65 crude product was tested for inhibitory effects on mushroom tyrosinase by using l-tyrosine as a substrate. Percentages of inhibition of mushroom tyrosinase were measured. Arbutin was used as the positive control. Calculated IC_50_ values for T65 crude product and arbutin were found to be 58.73 and 50.48 µg/mL, respectively (Figure 10). At a concentration of 300 µg/mL, T65 inhibited 84.98% of mushroom tyrosinase (Table S7). IC_50_ of the T65 culture extract was slightly lower than that of arbutin. However, the mushroom tyrosinase inhibition and melanin synthesis inhibition activities suggested that the culture extract of T65 was a non-cytotoxic strong whitening agent that could be used in the formulation of cosmeceuticals.

### 3.7. Analysis of Amino Acids

Analyses of free amino acids from T65 crude product were conducted by the Korea Polymer Testing and Research Institute (Seoul, South Korea). The total concentration of free amino acids obtained was 2328 mg/kg (Table S8). The amino acid with the highest concentration was valine (21.43%) and that with the least concentration was *α*-aminobutyric acid (0.81%). Characteristic composite amino acids are shown in Table S9. Eight different types of amino acids were determined as composite amino acids and the concentration of leucine was the highest (20.36%), whereas that of alanine was the lowest. Amino acids are protein substrates and are highly safe for humans, being commonly used in cosmetic ingredients [52,53]. Studies have shown that amino acids and their derivatives are an effective measure to suppress the signs of aging, oxidative stress, and melanogenesis [52,54,55,56,57].

Branched chain amino acids (BCAAs) (isoleucine, leucine, and valine) not only inhibit the melanogenesis but also restore the dermal collagen [52,58]. Specific amino acids, such as glycine and proline, are necessary to stimulate dermal tropocollagen synthesis [52]. In addition, free amino acids, such as alanine, proline, serine, glycine, methionine, tryptophan, lysine, and valine, lower the inflammation and oxidative stress by decreasing ROS production [56]. Aspartic acid and glutamic acid also possess antioxidant activities [59]. Furthermore, one or more residues of glutamic acid, lysine, aspartic acid, valine, alanine, proline, methionine, tryptophan, and phenylalanine improve the antioxidant activity [55,60]. Valine, leucine, serine, alanine, glycine, and glutamic acid have been reported to contribute to metal-chelating activity and tyrosinase-inhibition [57,61]. In T65 crude product, various residues of free and composite amino acids were detected that were capable for antioxidant, anti-aging, and anti-tyrosinase activities. Thus, bioactive material in T65 crude product may be composed of various peptides of more complete amino acids, reflecting its multi-functional activities. However, these mechanisms are complex and need further investigation.

### 3.8. Fatty Acids

Out of 37 types of fatty acids, only five (butyric acid, lauric acid, stearic acid, oleic acid, and palmitic acid) were detected by GC-FID. The highest concentration was of butyric acid (544 mg/kg; 54.67%). Fatty acid compositions of the extract are shown in Table S10. All the detected fatty acids in T65 crude product have been reported to have antioxidant activity [62,63,64,65,66].

### 3.9. Antimicrobial Activities

Five different types of solvent were used to extract the concentrated culture supernatant of strain T65. The inhibition zone (mean ± SD) using 15 µg of each extract are shown in Table S11. Among the five extracts, ethyl acetate extract had a higher inhibition for pathogenic bacteria. Inhibitory activity of ethyl acetate extract of strain T65 was greater than that of other solvent systems inhibiting the pathogens, including *P. acnes*, *S. aureus*, and *S. epidermidis* (Table S11). In addition, crude product from T65 inhibited *P. acnes*, *S. aureus*, *S. epidermidis, B. subtilis*, and *E. coli* (Figure S3; Table S12). These results suggest that strain T65 had a broad range of antimicrobial action and is able to inhibit Gram-positive as well as Gram-negative pathogens. Particularly, colonization of pathogenic bacteria, such as *P. acnes* and *S. epidermidis*, cause acne vulgaris (common skin disorder or a kind of infection of human skin that affects up to 80 % of people), and *S. aureus* causes nosocomial infection in the human face and skin [67,68]. Thus, the inhibition of these pathogens may help to reduce the colonization of such pathogens and to keep the skin healthy. In addition, inhibition of pathogens, such as *B. subtilis*, *E. coli*, and *P. aeruginosa*, may prevent the cosmetic emulsions from spoiling.

### 3.10. Mesoporous Silica Materials

Mesoporous material is a material containing pores with a diameter of 2 to 50 nm according to IUPAC (International Union of Pure and Applied Chemistry) nomenclature, and thus mesoporous silica material is a silica material that has pores that are 2–50 nm in diameter. This material was reported in the 1970s firstly in U.S. patents, but was not well noticed to the related fields, and thus similar material was independently researched and synthesized by Japan again in 1990 [69]. More detailed research was followed by Mobil Corporation Laboratories, and they named this material MCM (Mobil crystalline material), with examples such as MCM-41 or MCM-48, of which the mesopore diameter ranges from 2 to 6 nm [70,71]. Another research group in the University of California, Santa Barbara, succeeded in synthesizing a larger pore size (5–30 nm) mesoporous silica 6 years later, and they named it SBA (Santa Barbara amorphous material)-15 [72]. Mesopore silica material was designed at first to use as a molecular sieve due to its own structural character, but recently it has found a wide range of applications in drug delivery, catalyst, biosensor, energy storage, and so forth.

There are various differences in SBA-15 and MCM-41 mesoporous materials. However, the simplest way to distinguish these two is the silica wall thicknesses. The SBA-15 with a thicker silica wall has a more stable and rigid structure compared to that of MCM-41, which has thinner wall thickness. Moreover, the synthetic method of SBA-15 is simpler than that of MCM-41. Due to these differences, SBA-15 was selected to carry bioactive T65 crude product inside the mesopore of the silica material.

### 3.11. Synthesis of Mesoporous Silica Particles

After the calcination process was carried out under 200 °C for 2 h and 600 °C for 3 h continually, the resulting white silica mesoporous powder was obtained. SBA-15 of 8 nm pore were synthesized in this process. The particle sizes of SBA-15 were found to be 11.539–13.630 mm in diameter (Figure S4) and were determined by two different facilities: the Inha University Laboratory and the Changwon National University Laboratory. In addition, all the six samples in Figure S4 were synthesized by the same method but a different batch.

**Figure 11:**
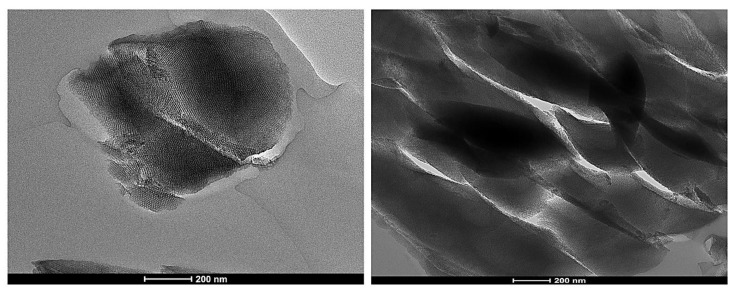
TEM (transmission electron microscopy) photomicrograph of synthesized mesoporous silica particles (SBA-15). Bar, 200 nm.

When the mixture was maintained at 60 °C and left for 4 days under static condition following the above method, SBA-15 of 5 nm pore was acquainted. Additionally, when the temperature was controlled at 120 °C, SBA-15 of 10 nm was synthesized. According to the results, mesopore size can be controlled depending on the aging process [73]. SBA-15 of 10 nm pore (Figure 11) was used for further processing because large pore mesoporous silica nanomaterials have better delivery actions for biomolecules than the narrow pore [74].

### 3.12. BET Surface Analyses

The pore volume (PV) value of synthesized mesoporous SBA-15 ranged from 35% to 49% (average 42%) depending on the synthetic conditions (Table 1). In addition, models of DFT (density functional theory) were used to determine the pore size distribution, and were also compared to relatively old BJH (Barret–Joyner–Halenda) [75].

In the environmental science field, pore volume (PV) is used to determine space volume in the particles. To determine the volume ratio more easily, the percentage unit is used rather than the decimal unit. For example, PV is written as 0.372607 (the unit is cm^3^/g); it can also be described as 37.3%. Compared to the normal spherical silica (SiO_2_, PV ≈0.2 on average) or spherical titania (TiO_2_, PV < 0.1), synthesized mesoporous SBA-15 shows a very efficient volume/mass ratio. Additionally, in this study, we achieved a porosity of 30%, which was established as a quantitative target.

### 3.13. Sustainable Performance Evaluation

To determine whether the mesoporous SBA-15 has a controlled-release ability, SBA-15 and the T65 crude product were combined together in DI water, methanol, acetonitrile, or ethylene glycol solvent. When SBA-15 and water were mixed, the solution became a slurry because of the coagulation between the silica surface and water. Methanol was a good solvent for both silica and T65 crude product. However, because of toxicity, methanol was substituted for acetonitrile. The particles for the T65 immersion ratio were determined by the PV value (Table S13). As shown in Table S13, the PV value of SBA-15 decreased after immersion with the T65 crude product. This showed that the T65 materials were immersed well inside the mesoporous silica particles.

To determine whether there was T65 inside the particle, T65 itself was investigated by HPLC (Figure S5). As shown by the data, there were two specific peaks (inside the red box) that were always visible in the case of the T65 culture extracts, even though the T65 preparation batch was changed. These two peaks had retention times of 10 min and 12 min at 230 nm. The filtrate sample was investigated in HPLC to determine whether T65 was present inside the embedded SBA-15. As shown in Figure S6, two specific peaks were observed near 10 min and 12 min. Furthermore, a small peak was observed near 18 min of retention time, which is very important to trace the T65 products that are time-released.

**Figure 12:**
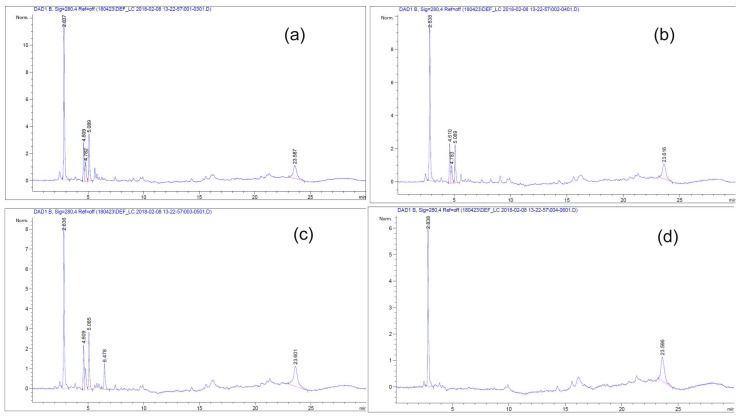
HPLC chromatogram of T65 embedded SBA-15 for sustainable control release test. (**a**) 0 day, (**b**) first day, (**c**) second day, and (**d**) third day.

Figure 12 represents the controlled release of the sustainable test of T65 embedded SBA-15 from 0 to 3 days. The specific peak of T65 was visible near 15 and 20 min from days 0 to 3. The T65 crude products were properly immersed and were gradually secreted over time. In addition, the silica mesoporous materials (SBA-15) proposed in this study could carry physiologically active substances in the T65 crude product and confirmed that the physiologically active substances were released in a sustained release manner after loading. Moreover, the silica mesoporous material proposed in this study could, in the future, be used as a good carrier to impregnate various functional materials and as a sustained-release material by supporting various physiologically active substances.

### 3.14. Cosmetic Formulation and Topical Application

The final cosmetic product (Figure S7) was formulated with various ingredients, as shown in Figure S8. The formulated cosmetic emulsion was between an essence and cream, having a certain amount of moisture with a low degree of oiliness. It had a feeling of oiliness just before the skin application. The final product of formulated cosmetic cream was designed on the basis of feeling and emulsifier-type cream. The formulation did not have a high degree of oiliness. After a certain period of time, the absorbency of the skin increased, leaving a moist feeling.

#### 3.14.1. Formulation Stability

The cosmetic samples were stored in the freezer, refrigerator, and at room temperature (37, 45, and 60 °C, respectively) for weeks. After 4 weeks, observations confirmed no separation or loose texture, and the stability of the cream was maintained. However, at 60 °C after the third and fourth week the formulated cream was observed to become slightly loose (Table S14).

#### 3.14.2. Clinical Trails

Formulated cosmetic cream was applied to 21 female volunteers. The age of volunteers was between 38 and 60 years (mean age ± SD, 46.43 ± 5.01). Determination of wrinkle improvement and whitening evaluation were conducted on the basis of skin roughness and skin brightness.

#### 3.14.3. Image Capturing and Processing

**Figure 13:**
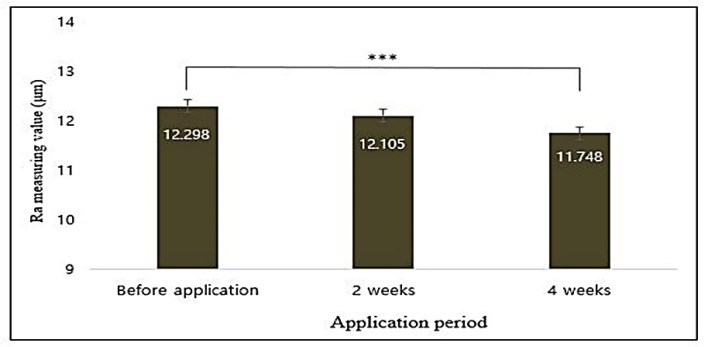
Correlation between application period of formulated cosmetics product and eye wrinkle average roughness values (Ra) analyzed using PRIMOS High Resolution. Data are given as mean ± SEM and statistical significance is shown by ANOVA (*** *p* < 0.001).

Face pictures of volunteers were captured by VISIA-CR. The roughness of captured images was analyzed using PRIMOS software (PRIMOS version 5.8E, Canfield Scientific, Inc., Parsippany-Troy Hills, NJ, USA) and brightness of images was analyzed using Chromameter CR-400. The three-dimensional measurement of PRIMOS was based on the computer-assisted calculation of the parallel stripes projected onto the skin according to the height difference of the skin surface. Using the 3D matching method, the corrugation images measured by PRIMOS were matched with the measured areas and the parameters through roughness analysis. Average roughness depth (Rmax) of the absolute values was the length from the centerline to the cross-section of the surface when drawing the centerline at the roughness height of the cross section. The results of analysis of the values of the eye wrinkle roughness using PRIMOS High Resolution showed that the Ra value was statistically significant (*p* < 0.001) compared to that after sample application and decreased by 4.656% after 4 weeks of application (Figure 13).

**Figure 14:**
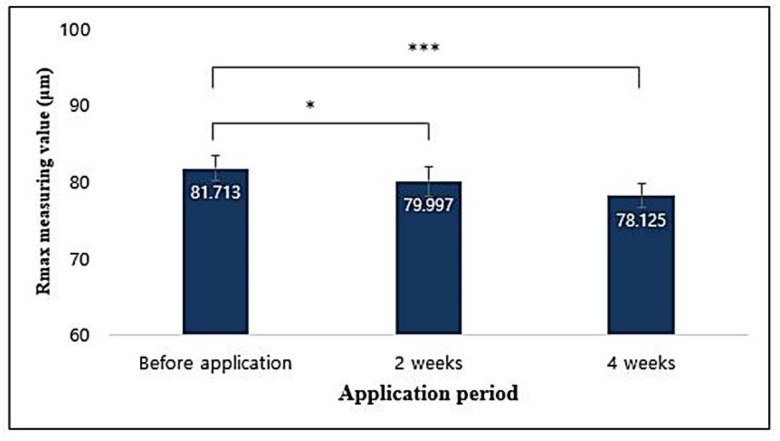
Correlation between application period of formulated cosmetics product and eye wrinkle roughness depth values (Rmax) analyzed using PRIMOS High Resolution. Data are given as mean ± SEM and statistical significance is shown by ANOVA (* *p* < 0.05; *** *p* < 0.001).

Rmax values were statistically significant (*p* < 0.05) as compared to those before application of the sample and decreased by 2.198% and 4.670% after 2 and 4 weeks of application, respectively (Figure 14). Therefore, it was considered that the T65 crude product complex functional cream helped to reduce the roughness of the eye folds after 2 and 4 weeks of application.

**Figure 15:**
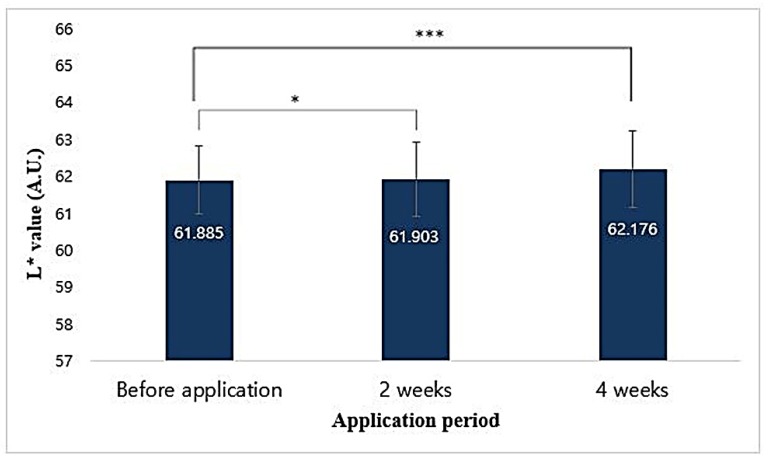
Correlation between application period of formulated cosmetics product and chromameter L* values. Data are given as mean ± SEM and statistical significance is shown by ANOVA (* *p* < 0.05; *** *p* < 0.001).

The L* value of the ball area using the chromameter was statistically significant (*p* < 0.001) compared with that before the application of the sample, and increased by 0.472% after 4 weeks of application (Figure 15). This result confirmed that the T65 crude product complex functional cream helped to improve skin brightness after 4 weeks of application.

## 4. Conclusions

Secondary bioactive materials extracted from the soil microorganisms are of interest for cosmetic applications owing to their antimicrobial, antioxidant, anti-wrinkle, skin whitening, and antimicrobial activities. This study describes the novel activity of ethyl acetate extract of strain T65 culture for the functional ingredients in cosmetic formulations. The T65 crude product was non-toxic to the different cell lines, such as HaCaT (human keratinocyte), RAW264.7 (macrophage cell), CCD-986Sk (human skin fibroblast cell), and B16F1 and B16F10 melanoma cells. In addition, T65 crude product showed upregulation of collagen type I synthesis, which is very important for reducing wrinkles on the skin. Furthermore, T65 crude product sufficiently inhibited the human pathogenic bacteria such as *B. subtilis*, *E. coli*, *P. acnes*, *S. aureus*, and *S. epidermidis*, which may help to prevent spoilage of the cosmetic product and colonization of pathogenic bacteria forming acne vulgaris on human skin. Moreover, T65 crude product inhibited the mushroom tyrosinase for whitening effect and porcine pancreatic elastase for anti-aging activities, and inhibited NO and scavenged DPPH radicals for antioxidant activities. Synthesis of mesoporous silica particles, SBA-15, proved that the T65 crude product would be effective in the cosmeceutical formulations with effective controlled release properties. Finally, in vivo application of formulated cosmetic product along with T65 crude product on the faces of volunteers proved the whitening and anti-wrinkle effect of T65. However, the chemistry of different bioactive compounds and the molecular mechanisms related to the different enzyme inhibition and pathogen inhibition activities remain to be discovered. In conclusion, our study strongly supported the idea that bacterial resources can be added to topically applied cosmetic products for whitening effect, anti-wrinkle formation, anti-oxidant effect, and antimicrobial activities.

## Figures and Tables

**Table 1:** Brunauer–Emmett–Teller (BET) analysis of SBA-15.

SN	Sample	BSA	PV	PS
1	SB0053-18	708.8383	0.382060	21.5598
2	SB0053-35	278.9649	0.367949	52.7593
3	SB0053-36	345.9479	0.485290	56.1114
4	SB0053-60	284.7321	0.372607	52.3449
5	SB0053-3	348.2327	0.489289	56.2025
6	SB0053-2	559.3040	0.423800	30.3096

SN: serial number; BSA: BET surface area (m^2^/g); PV: pore volume (cm^3^/g); PS: pore size (Å).

## Supplementary Materials

The following are available online at https://www.mdpi.com/2076-3921/9/4/278/s1, Figure S1: Neighbor–joining tree based on 16S rRNA gene sequences showing the phylogenetic position of strain T65 among closely related members of the family Streptomycetaceae., Figure S2: Evaluation of cell toxicity in HaCaT human keratinocyte cell line, Figure S3: Zone of inhibition of T65 crude product against human pathogens, Figure S4: Analysis of particle size of SBA, Figure S5: HPLC chromatogram of T65 extract, Figure S6: HPLC data of T65 embedded SBA-15, Figure S7: Final product of aesthetic product formulated using T65 crude product, Figure S8: List of ingredients used in the formulation of cosmetic product, Table S1: Components of reaction mixtures for DPPH free radical scavenging assays, Table S2: Total reaction mixture for elastase inhibition assays, Table S3: Total reaction mixture for for tyrosinase inhibition assays, Table S4: List of bacterial strains having potential activities in cosmeceuticals, Table S5: DPPH free radical scavenging activities of T65 crude product, Table S6: Porcine pancreatic elastase (PPE) inhibition activities of T65 crude product, Table S7: Mushroom tyrosinase inhibition activities of T65 crude product, Table S8: Free amino acids from ethyl acetate extract of strain T65, Table S9: Composite amino acids from T65 ethyl acetate extract, Table S10: Fatty acids from ethyl acetate extract of strain T65, Table S11: Zone of inhibition of T65 crude extract from five different solvent systems, Table S12: Zone of inhibition of purified crude T65 compounds against gram-positive and gram-negative pathogens, Table S13: Determination of T65 immersion using BET analyses, Table S14: Stability of formulated cream in different conditions and temperatures.
