# Supplementary material for: Development of Multifunctional Cosmetic Cream Using Bioactive Materials from Streptomyces sp. T65 with Synthesized Mesoporous Silica Particles SBA-15
Source: Antioxidants (Basel). 2020 Mar 26;9(4):278. doi: 10.3390/antiox9040278 (PMC7222176; doi:10.3390/antiox9040278)
Supplement: Supplementary file 1 [file antioxidants-09-00278-s001.pdf]

Supplementary Table 1. Components of reaction mixtures for DPPH free radical scavenging assays.

Total reaction mixture (180 μL)	Sample	0.1 M DPPH	Solvent	MtOH
Standard	–	90 μL	90 μL	–
Experiment	90 μL	90 μL	–	–
Control	90 μL	–	–	90 μL
Blank	–	–	90 μL	90 μL

Supplementary Table 2. Total reaction mixture for elastase inhibition assays.

**Total reaction mixture (150 μL)**	**Buffer**	**Sample (Solvent)**	**PPE**	**Substrate (Buffer)**
Standard	119.4 μL	(15 μL)	0.6 μL	15 μL
Experiment	119.4 μL	15 μL	0.6 μL	15μL
Control	119.4 μL	15 μL	0.6 μL	(15 μL)
Blank	119.4 μL	(15 μL)	0.6 μL	(15 μL)

Supplementary Table 3. Total reaction mixture for for tyrosinase inhibition assays.

**Total reaction mixture (150 μL)**	**Buffer**	**Sample (Solvent)**	**PPE**	**Substrate (Buffer)**
Standard	119.4 μL	(15 μL)	0.6 μL	15 μL
Experiment	119.4 μL	15 μL	0.6 μL	15μL
Control	119.4 μL	15 μL	0.6 μL	(15 μL)
Blank	119.4 μL	(15 μL)	0.6 μL	(15 μL)

Supplementary Table 4. List of bacterial strains having potential activities in cosmeceuticals.

Strains	Closest strain on the basis of 16S rRNA gene sequencing	Similarity (%)	GenBank accession
7Spe-2	*Brevibacillus formosus* DSM 9885^T	99.93	MN176423
Anti-1	*Bacillus acidiceler* CBD 119^T	98.57	MN176424
Anti-3	*Lysinibacillus xylanilyticus* DSM 23493^T	99.46	MN176425
Anti-5	*Bacillus acidiceler* CBD 119^T	99.38	MN176426
NHI-3	*Chitinophaga dinghnensis* DHOC24^T	96.96	MH817396
R11	*Dietzia cinnamea* IMMIB RIV-399^T	99.93	MH817397
R161	*Calidifontibacter indicus* PC IW02^T	97.71	KU881047
R301	*Streptomyces tanashiensis* LMG 20274^T	98.69	MH217561
R302	*Streptomyces roseolus* NBRC 12816^T	98.53	MH217563
R306	*Staphylococcus hominis* DSM 20328^T	99.86	MH817399
R308	*Streptomyces showdoensis* NBRC 13417^T	99.37	MH817400
R311	*Streptomyces gardneri* NBRC 12865^T	99.72	KX010107
R314	*Streptomyces zaomyceticus* NBRC 13348^T	99.86	KX010108
R316	*Streptomyces virginae* NBRC 12827^T	99.45	KX010109
R322	*Streptomyces scabiei* NRRL B-16523^T	99.93	MH817401
R351	*Streptomyces tanashiensis* LMG 20274^T	99.38	KX010110
R353	*Streptomyces gardneri* NBRC 12865^T	99.79	KX010111
R355	*Streptomyces xanthophaeus* NRRL B-5414^T	100	MH817402
R356	*Streptomyces amritsarensis* MTCC 11845^T	100	MH817403
R363	*Paenibacillus peoriae* DSM 8320^T	99.66	MH817404
R367	*Bacillus cereus* ATCC 14579^T	100	MH817405
R373	*Cellulosimicrobium funkei* ATCC BAA-886^T	99.93	MH817406
R379	*Streptomyces xanthocidicus* NBRC 13469^T	99.79	MH817407
R380	*Streptomyces exfoliatus* NRRL B-2924^T	99.86	MH817408
R386	*Streptomyces cinnamonensis* NBRC 15873^T	99.18	KX010112
R391	*Streptomyces neopeptinius* KNF 2047^T	99.21	MH817409
R397	*Streptomyces virginae* NBRC 12827^T	99.25	KX010113
R401	*Streptomyces tanashiensis* LMG 20274^T	99.65	MH817410
R407	*Bacillus acidiceler* CBD 119^T	99.32	MH817411

R434	*Actinokineospora soli* YIM 75948^T	98.54	KU881048
R438	*Bacillus thuringiensis* ATCC 10792^T	100	MH817412
R99	*Enterobacter asburiae* JCM6051^T	99.79	MH817398
RD-1-5	*Paenibacillus ginsengarvi* Gsoil 139^T	97.11	MH922828
RD-2-11	*Paenibacillus panacisoli* Gsoil 1411^T	99.73	MH922831
RD-2-2	*Streptomyces rubidus* 13C15^T	98.94	MH922829
RD-2-3	*Streptomyces rubidus* 13C15^T	99.86	MH922830
RD-2-37	*Spirosoma aerolatum* KACC 17939^T	96.36	MH922832
RD-2-6	*Streptomyces setonii* NRRL ISP-5322^T	100	MN181373
RD-3-1	*Streptomyces adustus* WH-9^T	99.93	MH922833
RD-3-11	*Streptomyces anulatus* NRRL B-2000^T	100	MH922838
RD-3-2	*Streptomyces collinus* NBRC 12759^T	99.3	MH922834
RD-3-20	*Streptomyces olivochromogenes* DSM 40451^T	99.93	MN181374
RD-3-5	*Paenibacillus taichungensis* BCRC 17757^T	99.8	MH922835
RD-3-6	*Aneurinibacillus soli* CB4^T	99.79	MH922836
RD-3-7	*Streptomyces sasae* JR-39^T	99.86	MH922837
RJ-1	*Virgibacillus proomii* LMG 12370^T	99.86	MN176427
RJ-10	*Enterococcus gallinarum* NBRC 100675^T	99.73	MN176459
RJ-10-10	*Bacillus aryabhattai* B8W22^T	100	MN176464
RJ-10-11	*Cellulomonas persica* JCM 18111^T	98.33	MN176465
RJ-10-13	*Acinetobacter lactucae* NRRL B-41902^T	99.79	MN176466
RJ-10-14	*Serratia marcescens* subsp. Sakuensis KRED^T	99.39	MN176467
RJ-10-16	*Serratia marcescens* subsp. Sakuensis KRED^T	99.66	MN176468
RJ-10-18	*Atlantibacter hermannii* NCTC12129	99.93	MN176469
RJ-10-20	*Pedobacter tournemirensis* TF5-37.2-LB10^T	99.29	MN176470
RJ-10-21	*Bacillus thuringiensis* ATCC 10792^T	100	MN176471
RJ-10-23	*Bacillus infantis* NRRL B-14911^T	99.86	MN176472
RJ-10-31	*Nocardioides caeni* MN8^T	99.31	MN176473
RJ-10-4	*Sanguibacter keddieii* DSM 10542^T	99.45	MN176460
RJ-10-6	*Serratia marcescens* subsp. Sakuensis KRED^T	99.66	MN176461
RJ-10-8	*Bacillus aryabhattai* B8W22^T	100	MN176462
RJ-10-9	*Bacillus aryabhattai* B8W22^T	100	MN176463
RJ-11-1	*Bacillus aryabhattai* B8W22^T	99.8	MN176474
RJ-11-2	*Bacillus thuringiensis* ATCC 10792^T	100	MN176475

RJ-12-2	*Microbacterium pseudoresistens* CC-5209^T	98.2	MN176476
RJ-15-1	*Bacillus megaterium* NBRC 15308^T	99.93	MN176477
RJ-15-2	*Psychrobacillus psychrodurans* DSM 11713^T	99.93	MN176478
RJ-15-3	*Bacillus acidiceler* CBD 119^T	99.86	MN176479
RJ-2	*Virgibacillus proomii* LMG 12370^T	99.86	MN176428
RJ-2-1	*Bacillus aryabhattai* B8W22^T	100	MN176429
RJ-2-2	*Bacillus thuringiensis* ATCC 10792^T	100	MN176430
RJ-2-3	*Bacillus toyonensis* BCT-7112^T	100	MN176431
RJ-3-1	*Bacillus idriensis* SMC 4352-2^T	99.79	MN176432
RJ-4-1	*Bacillus aryabhattai* B8W22^T	100	MN176433
RJ-4-2	*Chryseobacterium vietnamense* GIMN1.005^T	99.28	MN176434
RJ-5-4	*Chitinophaga caseinilytica* S-52^T	99.93	MN176435
RJ-5-5	*Bacillus wiedmannii* FSL W8-0169^T	100	MN176436
RJ-6	*Enterococcus gallinarum* NBRC 100675^T	99.86	MN176437
RJ-6-2	*Microbacterium foliorum* DSM 12966^T	99.65	MN176438
RJ-6-4	*Streptomyces stelliscabiei* NRRL B-24447^T	99.72	MN176439
RJ-6-7	*Chitinophaga deserti* XJ-2^T	99.72	MN176440
RJ-7	*Enterococcus mundtii* DSM 4838^T	99.12	MN176441
RJ-7-11	*Brevibacillus brevis* NBRC 15304^T	99.72	MN176449
RJ-7-13	*Stenotrophomonas* sp. POC10	99.66	MN176450
RJ-7-14	*Chryseobacterium geocarposphaerae* 91A-561^T	98.17	MN176451
RJ-7-2	*Brevibacillus formosus* DSM 9885^T	99.79	MN176442
RJ-7-3	*Microbacterium arabinogalactanolyticum* IFO 14344^T	99.29	MN176443
RJ-7-4	*Brevibacillus formosus* DSM 9885^T	99.79	MN176444
RJ-7-5	*Bacillus cereus* ATCC 14579^T	100	MN176445
RJ-7-6	*Brevibacillus formosus* DSM 9885^T	99.86	MN176446
RJ-7-7	*Brevibacillus formosus* DSM 9885^T	99.86	MN176447
RJ-7-9	*Brevibacillus formosus* DSM 9885^T	99.93	MN176448
RJ-8	*Serratia marcescens* ATCC 13880^T	99.86	MN176452
RJ-8-1	*Serratia marcescens* ATCC 13880^T	99.86	MN176453
RJ-8-3	*Enterobacter kobei* JCM 8580^T	98.36	MN176454
RJ-8-4	*Bacillus acidiceler* CBD 119^T	98.77	MN176455
RJ-8-5	*Bacillus toyonensis* BCT-7112^T	99.86	MN176456
RJ-8-6	*Bacillus acidiceler* CBD 119^T	98.71	MN176457

RJ-9-2	*Bacillus proteolyticus* TD42^T	99.86	MN176458
T1317-0309	*Streptomyces lannensis* TA4-8^T	99.99	MF784398
T327	*Streptomyces glebosus* CGMCC 41873^T	99.42	KX602661
T65	*Streptomyces bungoensis* NBRC 15711^T	99.65	KX010114
T811	*Streptomyces phaeopurpureus* NRRL B-2260^T	99.86	KX602662

Supplementary Table 5. DPPH free radical scavenging activities of T65 crude product

	**Inhibition (%)**					IC_{50} μg/mL
Inhibitor	300 μg/mL	100 μg/mL	50 μg/mL	10 μg/mL	2 μg/mL	
T65 crude product	89.98 ± 2.56	85.66 ± 2.74	81.04 ± 1.98	68.66 ± 2.83	22.02 ± 2.21	6.31 ± 0.94
Vitamin C	93.65 ± 1.01	89.69 ± 1.31	85.26 ± 2.01	73.98 ± 1.60	24.29 ± 1.10	5.01 ± 0.89

Supplementary Table 6. Porcine pancreatic elastase (PPE) inhibition activities of T65 crude product.

	**Inhibition (%)**				
Inhibitor	10 μg/mL	50 μg/mL	100 μg/mL	300 μg/mL	IC_{50} μg/mL
T65 crude product	50.29 ± 1.05	57.16 ± 2.14	64.07 ± 2.88	87.57 ± 3.03	14.68 ± 1.11
Oleanolic acid	52.32 ± 1.31	65.73 ± 1.9	76.38 ± 2.01	92.85 ± 2.6	10.19 ± 0.98

Supplementary Table 7. Mushroom tyrosinase inhibition activities of T65 crude product.

	**Inhibition (%)**				IC_{50} μg/mL
Inhibitor	10 μg/mL	50 μg/mL	100 μg/mL	300 μg/mL	
T65 crude product	13.23 ± 0.98	45.29 ± 2.12	64.32 ± 3.11	84.98 ± 2.98	58.73 ± 1.23
Arbutin	13.31 ± 0.73	49.23 ± 1.13	68.96 ± 2.24	91.25 ± 2.1	50.48 ± 1.68

Supplementary Table 8. Free amino acids from ethyl acetate extract of strain T65.

SN	Amino acids	Cocentration (mg/Kg)
1	4-Hydroxyproline	ND
2	Alanine	241
3	α-Aminoadipic acid	49
4	α-Aminobutyric acid	19
5	α-Aminopimelic acid	ND
6	allo-Isoleucine	ND
7	Asparagine	39
8	Aspartic acid	35
9	β-Aminobutyric acid	ND
10	Cystathionine	ND
11	Cysteine	ND
12	Glutamic acid	65
13	Glutamine	ND
14	Glycine	20
15	Glycine-proline	ND
16	Histidine	ND
17	Hydroxylysine	ND
18	Isoleucine	320
19	Leucine	395
20	Lysine	21
21	Methionine	88
22	Noraline	ND
23	Ornithine	ND
24	Phenylalanine	144
25	Proline	87
26	Proline-hydroxyproline	ND
27	Sarcosine	ND
28	Serine	89

29	Thioproline	ND
30	Threonine	116
31	Tryptophan	101
32	Valine	499
	**Total**	**2328**

Supplementary Table 9. Composite amino acids from T65 ethyl acetate extract.

SN	Amino acids	Concentration (mg/Kg)
1	4-Hydroxyproline	ND
2	Alanine	729
3	Aspartic acid	ND
4	Cystine	ND
5	Glutamic acid	7501
6	Glycine	1561
7	Histidine	ND
8	Hydroxylysine	ND
9	Isoleucine	4319
10	Leucine	10251
11	Lysine	ND
12	Methionine	ND
13	Noraline	ND
14	Phenylalanine	9667
15	Proline	ND
16	Serine	6540
17	Threonine	ND
18	Valine	9774
	**Total**	**50342**

Supplementary Table 10. Fatty acids from ethyl acetate extract of strain T65.

SN	Fatty acids	Concentration (mg/Kg)
1	Butyric acid	544
2	Arachidic acid	ND
3	Arachidonic acid	ND
4	Behenic acid	ND
5	Capric acid	ND
6	Caproic acid	ND
7	Caprylic acid	ND
8	Cis–10–Heptadecenoic acid	ND
9	Cis–10–Pentadecenoic acid	ND
10	Cis–11,14,17–Eicosatrienoic acid	ND
11	Cis-11,14–Eicosadienoic acid	ND
12	Cis–11–Eicosenoic acid	ND
13	Cis–13,16–Docosadienoic acid	ND
14	Cis–4,7,10,13,16,19–Docosahexaenoic acid	ND
15	Cis–5,8,11,14,17–Eicosapentaenoic acid	ND
16	Cis–8,11,14–Eicosatrienoic acid	ND
17	Elaidic acid	ND
18	Erucic acid	ND
19	Heneicosanoic acid	ND
20	Heptadecanoic acid	ND
21	Lauric acid	193
22	Lignocceric acid	ND
23	Linoleic acid	ND
24	Linolelaidic acid	ND
25	Linolenic acid	ND
26	Myristic acid	ND
27	Myristoleic acid	ND
28	Nervonic acid methyl ester	ND
29	Oleic acid	80

30	Palmitic acid	122
31	Palmitoleic acid	ND
32	Pentadecanoic acid	ND
33	Stearic acid	56
34	Tricosanoic acid	ND
35	Tridecanoic acid	ND
36	Undecanoic acid	ND
37	γ–Linolenic acid	ND
	**Total**	**995**

Supplementary Table 11. Zone of inhibition of T65 crude extract (15 μg) from five different solvent systems.

	Inhibition (mm)		
Solvent	*S. aureus*	*S. epidermidis*	*P. acnes*
Hexane	11.33 ± 0.58	10.33 ± 0.58	12.33 ± 0.58
Di-ethyl ether	11.33 ± 0.59	10.33 ± 0.59	12.0 ± 1.0
Di-chloromethane	15.0 ± 1.0	14.33 ± 0.60	15.67 ± 0.58
Chloroform	11.33 ± 1.15	11.33 ± 0.58	12.33 ± 0.58
Ethyl acetate	17.33 ± 0.58	20.33 ± 0.58	22.33.0 ± 1.15

Supplementary Table 12. Zone of inhibition of purified crude T65 compounds (15 μg) against gram-positive and gram-negative pathogens.

Strains	Inhibition (mm)
Bacillus subtilis KEMB 51201-001^{T}	12.33 ± 0.58

Escherichia coli KEMB 212-234^T	14.67 ± 0.58
Staphylococcus aureus KEMB 7301-069^T	17.33 ± 0.58
Staphylococcus epidermidis KACC 13234^T	20.33 ± 0.58
Propionibacterium acnes KCTC 3314^T	22.33.0 ± 1.15
Pseudomonas aeruginosa KACC 10185^T	12.0 ± 1.0

Supplementary Table 13. Determination of T65 immersion using BET analyses

STS-15 (treated with/without T65)		PV value (%)
Without T65 crude product		64.12
	1st run	55.44
	2nd run	50.1
With T65 crude product	3rd run	52.3

Supplementary Table 14. Stability of formulated cream in different conditions and temperatures

	**Condition of formulation**			
Temperature	1st week	2nd week	3rd week	4th week
Freezer	Good	Good	Good	Good
Refrigerator	Good	Good	Good	Good
Room Temperature	Good	Good	Good	Good
37 ºC	Good	Good	Good	Good
45 ºC	Good	Good	Good	Good

60 ºC	Good	Good	Loosen	Loosen

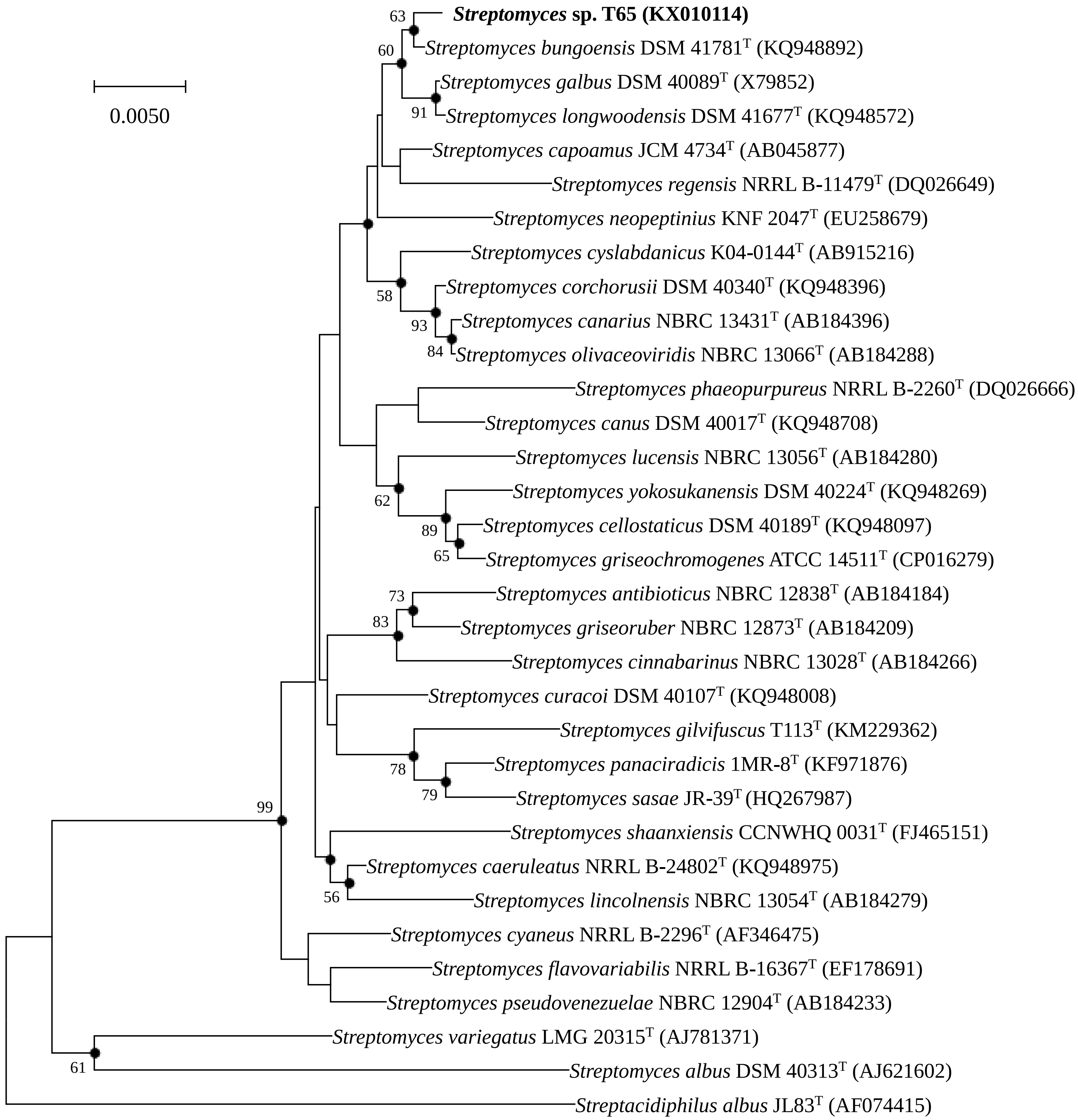

Supplementary Figure S1. Neighbor–joining tree based on 16S rRNA gene sequences showing the phylogenetic position of strain T65 among closely related members of the family *Streptomycetaceae*. Filled circles indicate nodes recovered by all three treeing

methods (neighbor–joining, maximum–likelihood, and maximum–parsimony). The numbers at the nodes indicate the percentage of 1000 bootstrap replicates yielding this topology; only values >50 % are shown. *Streptacidiphilus albus* JL83^{T} was used as an out-group. GenBank accession numbers are given in parentheses. Bar, 0.005 substitutions per nucleotide position.

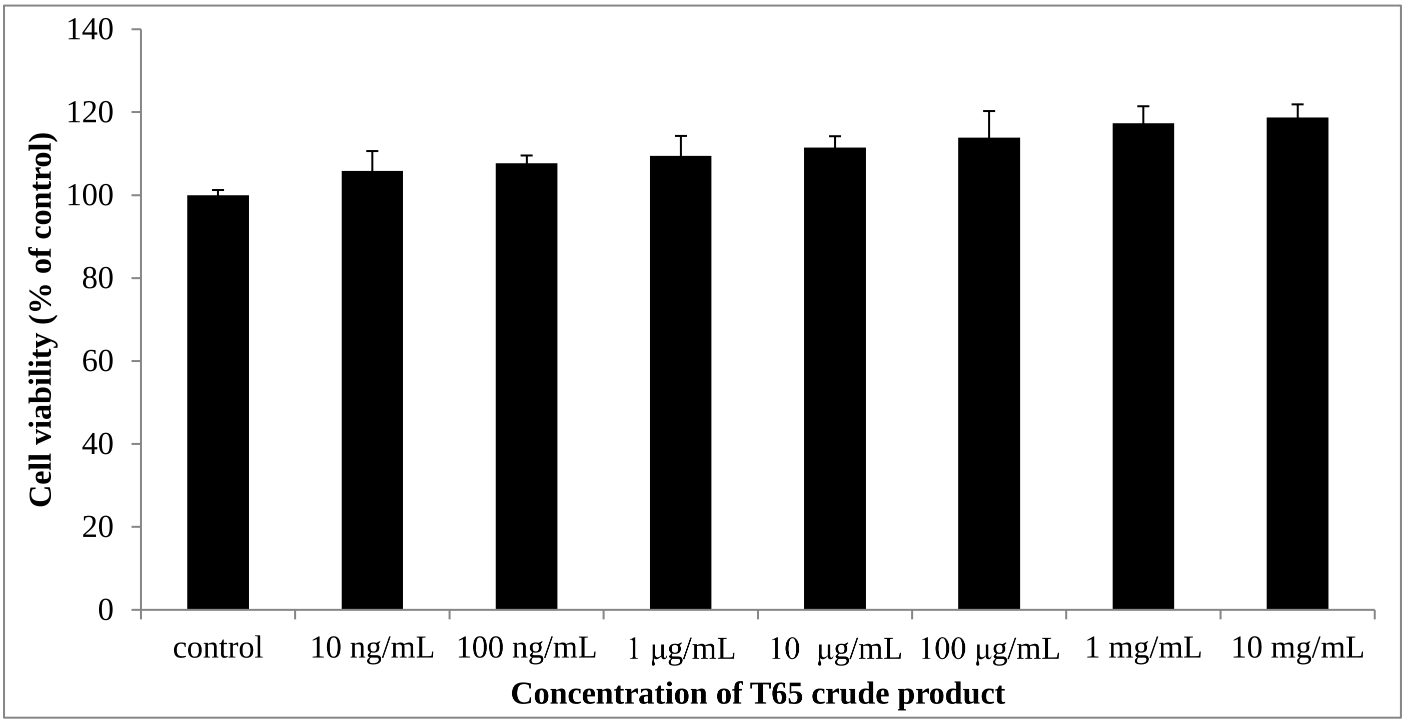

Supplementary Figure S2. Evaluation of cell toxicity in HaCaT human keratinocyte cell line.

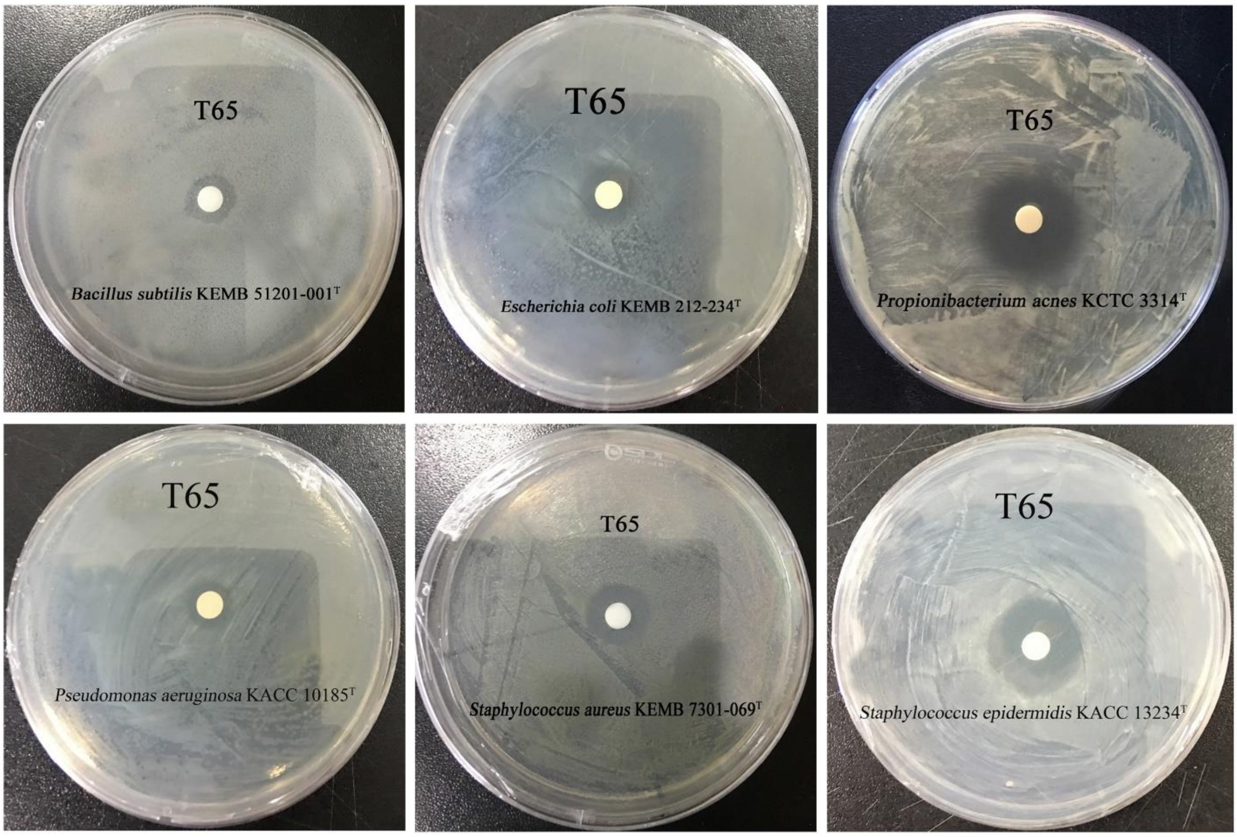

Supplementary Figure S3. Zone of inhibition of T65 crude product against human pathogens.

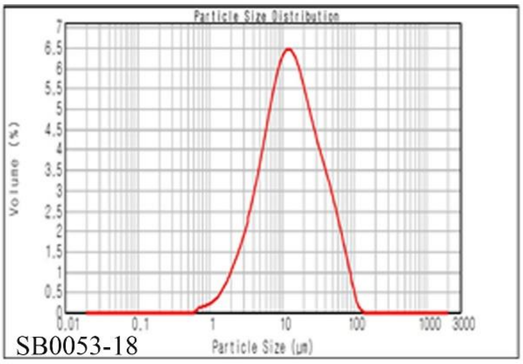

SB0053-18

d(0.5): 13.222

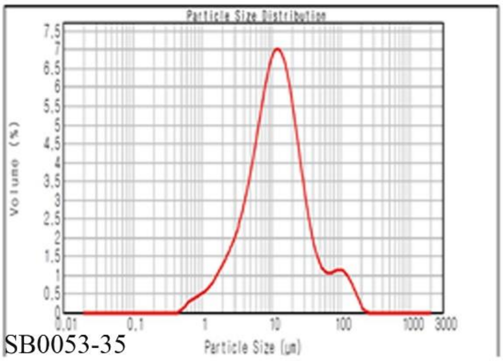

SB0053-35

d(0.5): 11.885

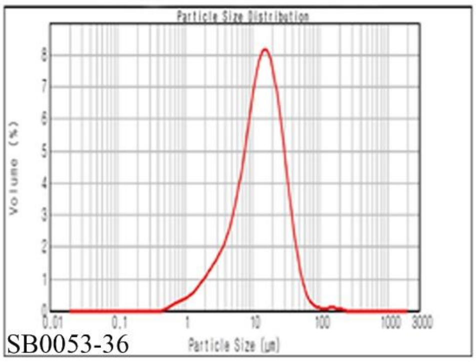

SB0053-36

d(0.5): 13.630

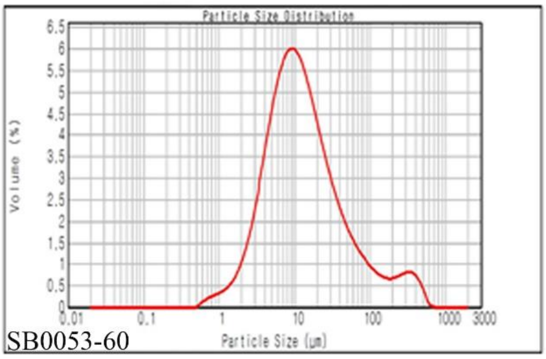

SB0053-60

d(0.5): 11.697

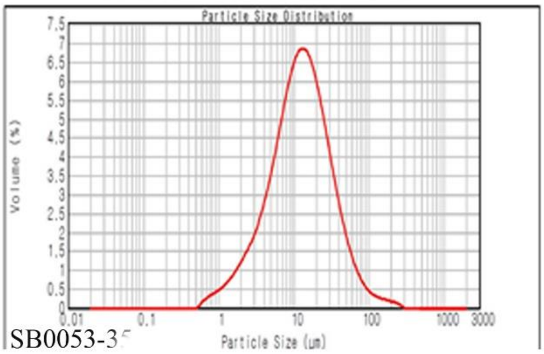

SB0053-3[illegible]

d(0.5): 12.523

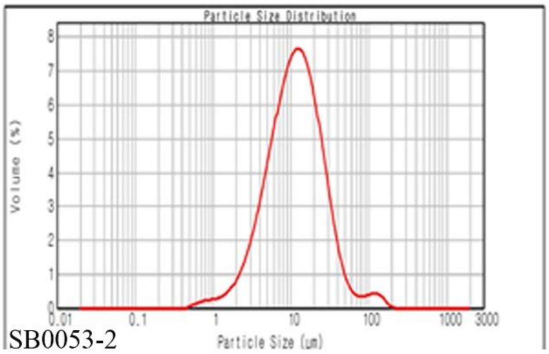

SB0053-2

d(0.5): 11.539

Supplementary Figure S4. Analysis of particle size of SBA-15. d(0.5), diameter of silica particle.

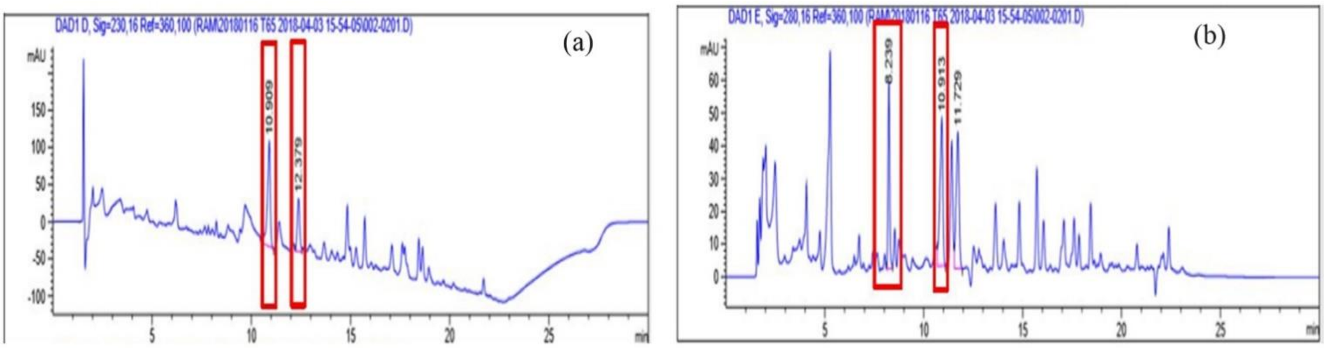

Supplementary Figure S5. HPLC chromatogram of T65 extract. a), 230 nm; b), 280 nm.

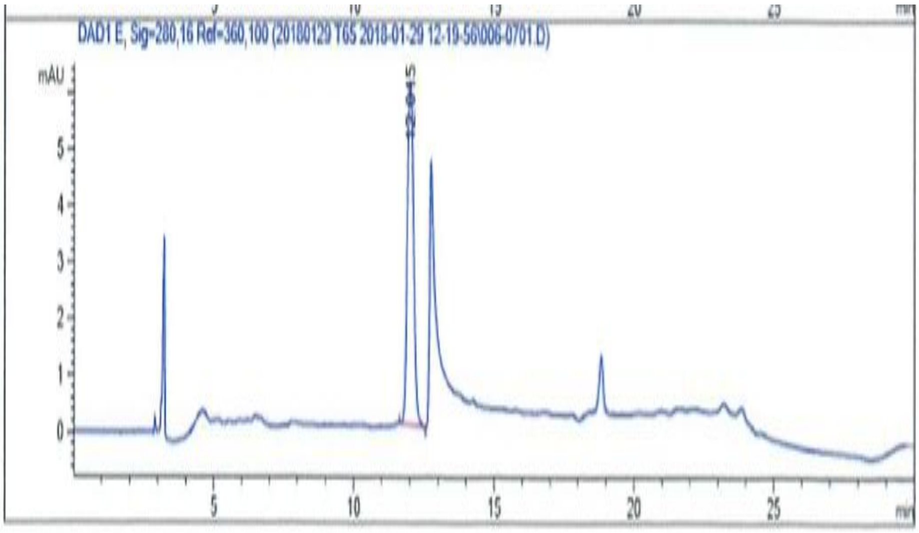

Supplementary Figure S6. HPLC data of T65 embedded SBA-15 (after treatment; 280 nm).

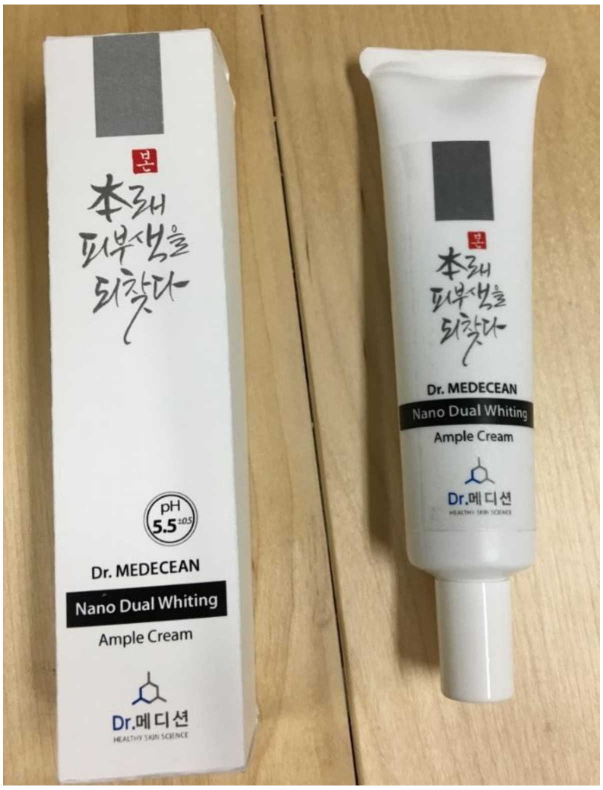

Supplementary Figure S7. Final product (40 g) of aesthetic product formul ated using T65 crude product.

Ammonium acryloyldimethyltaurate/vp copolymer, argan kernal oil, argan leaf extract, butylene glycol, butyryl alcohol, caprylyl glycol, carbomer, cetearyl alcohol, citric acid, cocoglycerides, copper tripeptide-1, dicaprylyl carbonate, dimethicone, dipropylene glycol, disodium iodide, ethanol, ethylhexylglycerin, genistein, glycerin, glyceryl stearate, hydrogenated polyisobutene, lactose, lecithin, p-anisic acid, pea extract, PEG-100 stearate, pentylene glycol, phenoxyethanol, phospholipid, polysorbate 20, polysorbate 80, propylheptyl caprylate, purified water, shea butter, slipper wood leaf oil, sodium citrate, sodium cocoyl glutamate, sodium dehydroacetate, sodium hyaluronate, sodium polyacrylate, sorbic acid, sucrose dilaurate, sucrose polystearate, T65 embedded SBA-15, t-butyl alcohol, tocopheryl acetate, vinyl alcohol, whey protein, and xanthan gum.

Supplementary Figure S8. List of ingredients used in the formulation of cosmetic product.
